# Effects of diversity on thermal niche variation in bird communities under climate change

**DOI:** 10.1038/s41598-022-26248-1

**Published:** 2022-12-17

**Authors:** Emma-Liina Marjakangas, Andrea Santangeli, Alison Johnston, Nicole L. Michel, Karine Princé, Aleksi Lehikoinen

**Affiliations:** 1 Finnish Museum of Natural History, University of Helsinki, PO Box 17, 00014 Helsinki, Finland; 2 Research Centre for Ecological Change, Organismal and Evolutionary Biology Research Programme, University of Helsinki, Viikinkaari 1, PO Box 65, 00014 Helsinki, Finland; 3 FitzPatrick Institute of African Ornithology, DST-NRF Centre of Excellence, University of Cape Town, Cape Town, South Africa; 4 Cornell Lab of Ornithology, 159 Sapsucker Woods Road, Ithaca, NY 14850 USA; 5 National Audubon Society, 225 Varick St, New York, NY 10014 USA; 6 UMR 7204 Centre d’Ecologie et des Sciences de la Conservation (CESCO), Muséum national d’histoire naturelle, 43, rue Buffon, 75005 Paris, France; 7 Department of Forest and Wildlife Ecology, University of Wisconsin-Madison, Madison, WI 53706 USA

**Keywords:** Biodiversity, Climate-change ecology, Community ecology, Ecological modelling, Macroecology

## Abstract

Climate change alters ecological communities by affecting individual species and interactions between species. However, the impacts of climate change may be buffered by community diversity: diverse communities may be more resistant to climate-driven perturbations than simple communities. Here, we assess how diversity influences long-term thermal niche variation in communities under climate change. We use 50-year continental-scale data on bird communities during breeding and non-breeding seasons to quantify the communities’ thermal variability. Thermal variability is measured as the temporal change in the community’s average thermal niche and it indicates community’s response to climate change. Then, we study how the thermal variability varies as a function of taxonomic, functional, and evolutionary diversity using linear models. We find that communities with low thermal niche variation have higher functional diversity, with this pattern being measurable in the non-breeding but not in the breeding season. Given the expected increase in seasonal variation in the future climate, the differences in bird communities’ thermal variability between breeding and non-breeding seasons may grow wider. Importantly, our results suggest that functionally diverse wildlife communities can mitigate effects of climate change by hindering changes in thermal niche variability, which underscores the importance of addressing the climate and biodiversity crises together.

## Introduction

Climate change affects biodiversity by impacting individual species^1^, pairwise interactions^2^, and entire communities^3^. Climate change can affect whole ecological communities directly by increasing the ratio of warm- versus cold-dwelling species^3–7^, and/or indirectly by altering species’ interactions and resources^8^. However, the exact processes that regulate these community-level responses to climate change are poorly understood.

**Figure 1:**
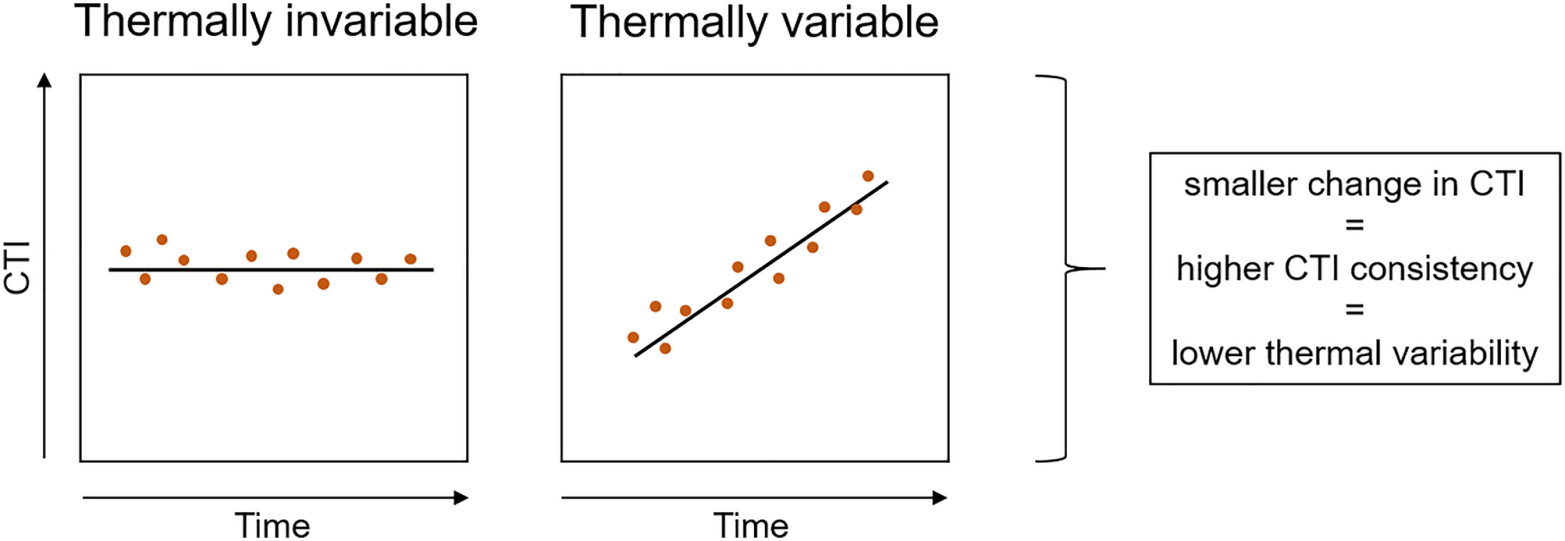
Graphical illustration of the concept of thermal variability. The coloured circles represent values of hypothetical community-level properties (here, community temperature index CTI) over time. The communities are less thermally variable if there is no clear long-term trend in the community property and more thermally variable if there is a clear long-term trend in the community property.

One way to measure the climate change response of communities is thermal variability^3,9^. Temporal variability in the thermal niche of the community can be measured as the temporal trend in the average thermal niche (i.e., temperature preference) of its species, such that the invariability in the average thermal niche indicates that a community is resisting temperature-related compositional changes under climate change^3,9^ (Fig. 1). We measure thermal variability using the community temperature index (CTI). CTI is a metric reflecting the thermal niche signature of the community, calculated as the average thermal niche of all species in the community (species-specific temperature indices, STI) weighted by species’ abundance^3,9,10^. STI represents long-term average temperature within the range extent of the species for a given season. Thermal variability is a useful measure, because climate change poses a directional long-term, rather than a single perturbation pressure to ecosystems. Given that climate change poses such a long-term pressure, our measure of thermal variability should be able to capture time lags in community properties. For example, the responses of species with slow life histories are likely to appear non-existent over short time scales. We considered a community as thermally invariable when mean CTI was consistent across years (Fig. 1).

The effects of climate change on ecosystems may be buffered by high biodiversity in species’ communities^11,12^. Diverse communities may be better able to resist compositional changes induced by climate because their species can better complement each other’s roles functionally and over time^13–15^. The diversity of a community can be measured in a myriad of ways, covering the taxonomic (e.g., species richness), functional (e.g., functional trait space dispersion), and evolutionary (e.g., phylogenetic diversity) dimensions of diversity^16–18^, all of which may reflect different intrinsic properties of the ecosystem^19^ (Table 1). Different diversity dimensions may be relevant for communities’ climate change responses in different contexts, for example depending on environmental conditions^19^. For example, high diversity and overall biomass tend to increase community stability under disturbance, such as droughts that are expected to increase with climate change^20^. Diverse communities can also hinder invasions of new species^21,22^ as niche space for new species may not exist in a functionally saturated community. Moreover, higher species’ specialization (in terms of abiotic and biotic niche breadth) can result in increased niche packing and higher diversity^23,24^, ensuring ecosystem functioning. In this paper, we consider the taxonomic, functional (trait-based), and evolutionary (phylogeny-based) dimensions of community diversity.

**Table 1:** List of hypotheses for the diversity-thermal variability relationship.

Diversity dimension	Model	Hypothesis	Ecological context	Sources
Taxonomic	Thermal variability ~ Number of species	Communities with more species have less variability in their average thermal niche over time	Species-rich communities include temporally and functionally complementary species that respond asynchronically to climate change	^11–13^
Functional	Thermal variability ~ Functional richness	Communities covering a larger functional space have less variability in their average thermal niche over time	Communities covering a larger niche space ensure functionality and prevent invasions of new species	^11,12,21,22^
Functional	Thermal variability ~ Functional dispersion	Functionally more dispersed communities have less variability in their average thermal niche over time	Communities with more evenly distributed functional niche space ensure functionality and prevent invasions of new species	^11,12,21,22^
Functional	Thermal variability ~ Community weighted mean of diet diversity	Communities with higher degree of diet generalism have less variability in their average thermal niche over time	Communities with higher degree of diet generalism adjust to climate-induced changes in food resources via interaction rewiring	^39,86^
Functional	Thermal variability ~ Community weighted mean of habitat niche breadth	Communities with higher degree of habitat generalism have less variability in their average thermal niche over time	Communities with higher degree of habitat generalism adjust to climate-induced changes in habitat via species-level responses	^72,86^
Functional	Thermal variability ~ Community weighted mean of thermal niche breadth	Communities with higher degree of thermal niche generalism have less variability in their average thermal niche over time	Communities with wider average temperature tolerance adjust to climate-induced changes in environmental conditions	^86^
Functional	Thermal variability ~ Community weighted mean of vertebrates in diet	Communities involving more top predators have less variability in their average thermal niche over time	Communities with higher degree of top predators have longer food chains, more complex network structure and more functional complementarity among species	^2,87,88^
Evolutionary	Thermal variability ~ Phylogenetic diversity	Communities with evolutionary more distinct lineages have less variability in their average thermal niche over time	Phylogenetically diverse communities have higher evolutionary potential to adapt and respond to changes in environmental conditions	^11,12^

The expected effect of each diversity measure on thermal variability is specified in column ‘Hypothesis’. For more details on the model structures, see Methods.

In the climate change context, both the thermal niche trends and the community diversity have been studied separately^10,25,26^, but the large-scale relationship between diversity and thermal variability has not yet been explored. So far, few studies have assessed the effects of diversity on the compositional responses of communities in the context of climate change (but see Catano et al.^27^). In particular, patterns of communities’ response to climate change can vary with spatial scale^28^ and therefore generalizations of communities’ climate change responses at a large spatial scale cannot be made based on local scale studies. To investigate the effect of diversity on thermal variability under climate change, long-term and large-scale community monitoring data of species’ abundances are needed. Such data can reveal patterns of populations’ and communities’ temporal variability that are known to vary with time scale^29^. Importantly, given the different forces acting across the annual cycle of animals, breeding and non-breeding season data are needed to disentangle potential inter-seasonal differences in climate-driven community changes^30^. Such comprehensive data are rare, but their use can increase our understanding of, and potential to alleviate, climate change effects on community compositions.

Here, we use comprehensive long-term large-scale datasets on bird communities surveyed during 1966–2016 in the breeding^31^ and non-breeding season^32^ in North America. Bird communities provide a highly suitable study system because birds are known to respond to climate change with range and phenology shifts^4,10,33^. Moreover, there are extensive systematic data of birds that provide a good sample of the whole bird community, which is essential for studies like ours that focus on the community composition. We explore how community diversity influences the climate change responses of bird communities across seasons. We ask: (1) Does community diversity affect the thermal variability of bird communities under climate change? and (2) Does the effect of diversity on bird communities’ thermal variability differ between breeding and non-breeding seasons? Based on the hypothesis of diversity-stability relationship in earlier studies^11,12^, we predict that diverse communities are less thermally variable and have less climate-driven structural changes, thus exhibiting less pronounced responses to climate change than simple communities (Table 1). We also predict that functional diversity has the strongest effect on thermal variability, because functional diversity has been found to predict ecosystem functioning in general^34^. Moreover, we predict that the effect of diversity on thermal variability differs between seasons because climate largely influences reproduction-related factors during breeding season and on survival-related factors during non-breeding season^35,36^. More specifically, species are less likely to shift their breeding grounds^35,36^, and climate change effects on CTI have been more evident in the non-breeding season^10,30,37^. Therefore, we expect the composition of the breeding season communities to be less thermally variable and less influenced by the overall diversity than that of the non-breeding season communities.

## Results

### Degree of thermal variability

**Figure 2:**
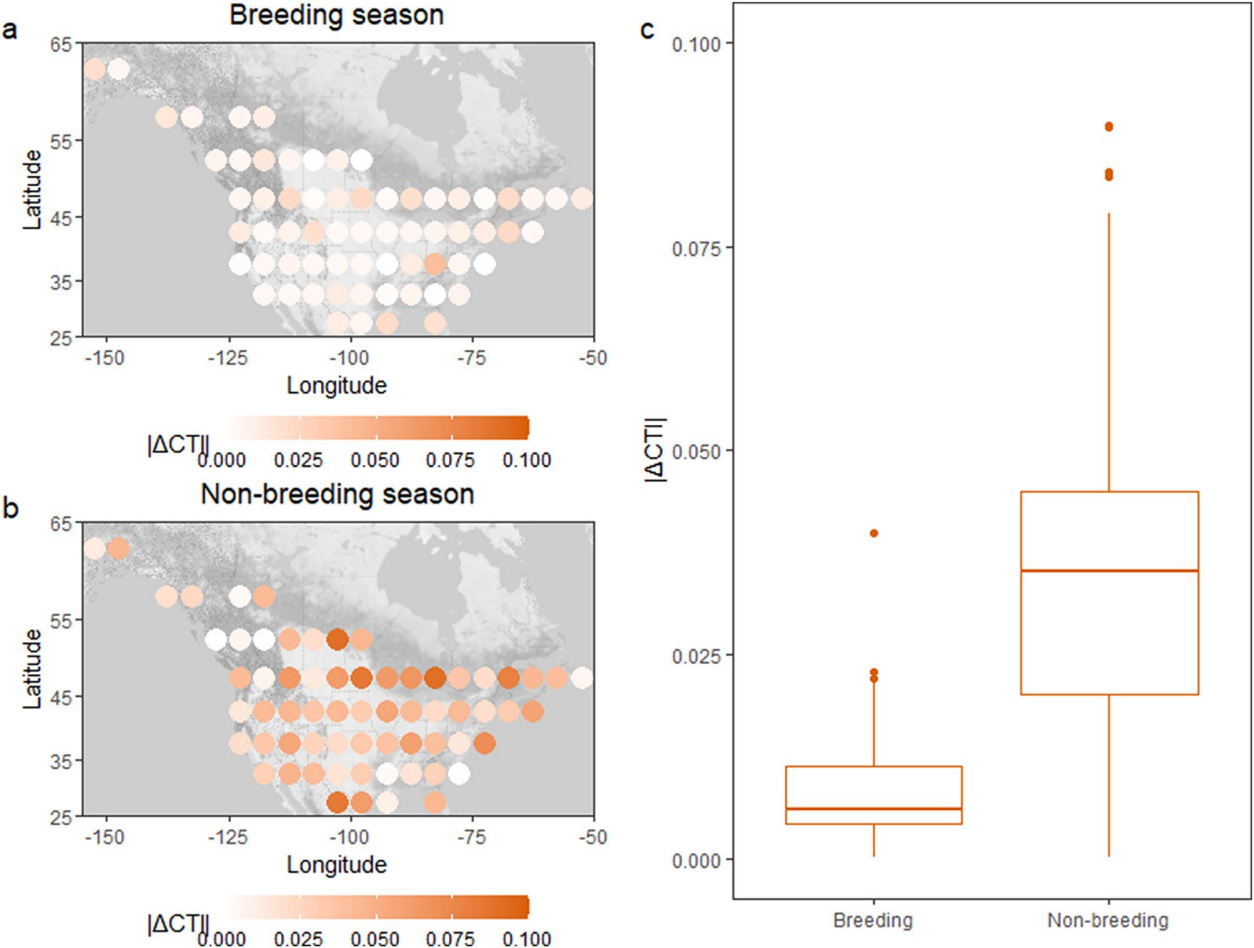
Spatial and seasonal variation in thermal variability. Grid cells of 5° × 5° were considered as study units (N = 65) across North America. Panels (**a**) and (**b**) illustrate the temporal trend in community temperature index (|∆CTI|; thermal variability) in breeding and non-breeding seasons, respectively. Panel (**c**) illustrates the seasonal difference in thermal variability values. In total, 586 and 698 species were included in the breeding and non-breeding season data analyses, respectively (for full species lists, see Appendix S2). The colour gradients show the thermal variability measure variation among grid cells. Figure was produced using R software (Version 3.5.3. URL: http://www.r-project.org/).

We found spatial variation in the thermal variability of bird communities across the study area in both seasons (Fig. 2a,b). Bird communities were on average less thermally variable (i.e., smaller change in CTI over time) in the breeding than in the non-breeding season (mean_breeding_ = 0.009, mean_non-breeding_ = 0.036, t = − 9.33, *p* < 0.001; Fig. 2c).

### Diversity-thermal variability relationships

**Figure 3:**
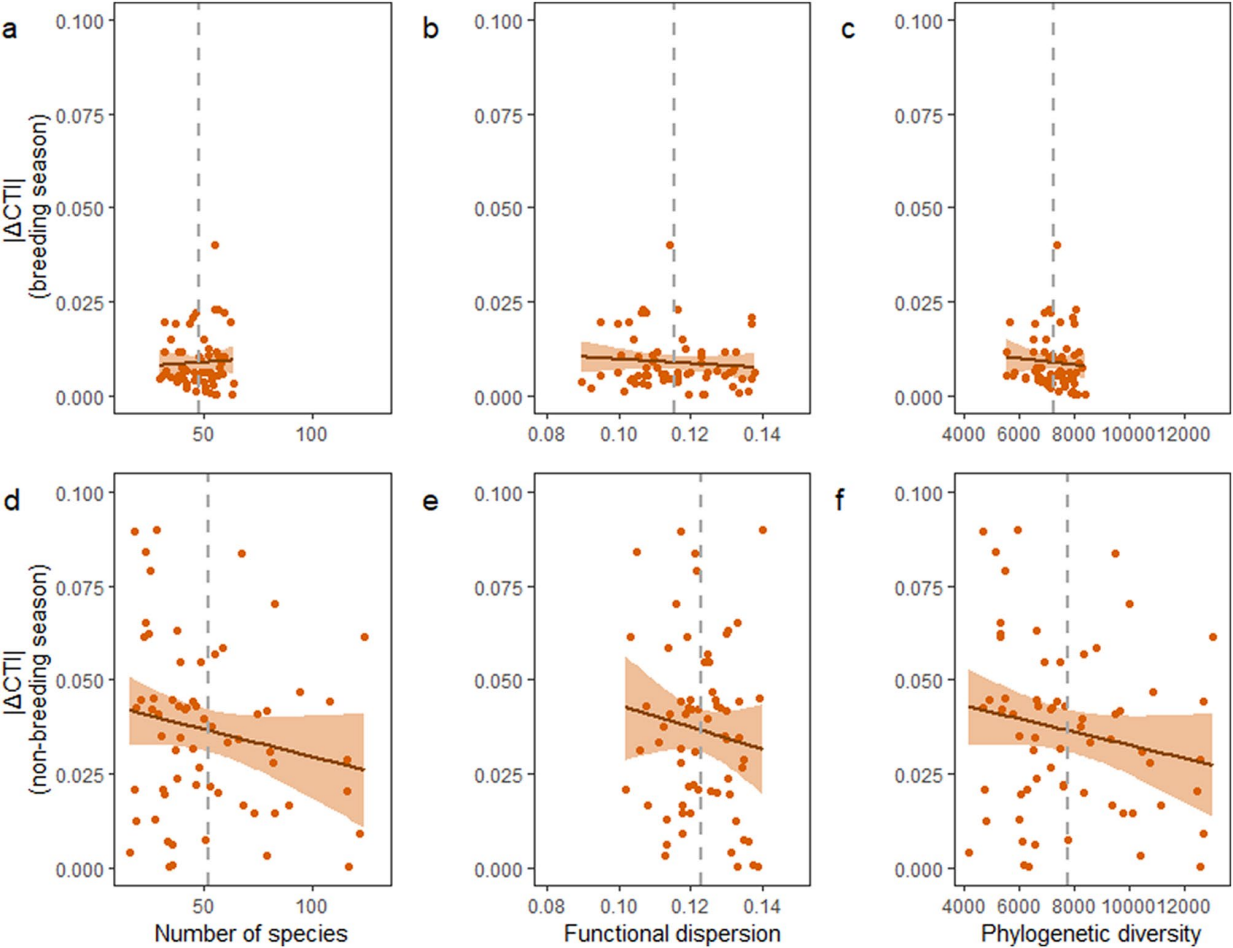
Examples of diversity-thermal variability relationships in breeding and non-breeding seasons. The raw linear relationships with measures of thermal variability and of taxonomic, functional, and evolutionary diversity for each season are illustrated. Thereby, panels (**a**) and (**d**) illustrate the temporal change in community temperature index (|∆CTI|) as a function of the number of species, panels (**b**) and (**e**) illustrate |∆CTI| as a function of the functional dispersion, and panels (**c**) and (**f**) illustrate |∆CTI| as a function of the phylogenetic diversity within the community. The mean of each diversity measure across grid cells is indicated with a grey dashed vertical line. Note that the effect of temperature trend or the spatial correlation structure are not accounted for in the model structures of the illustrated relationships (for full model statistics, see Appendix S1: Table S3). Figure was produced using R software (Version 3.5.3. URL: http://www.r-project.org/).

**Figure 4:**
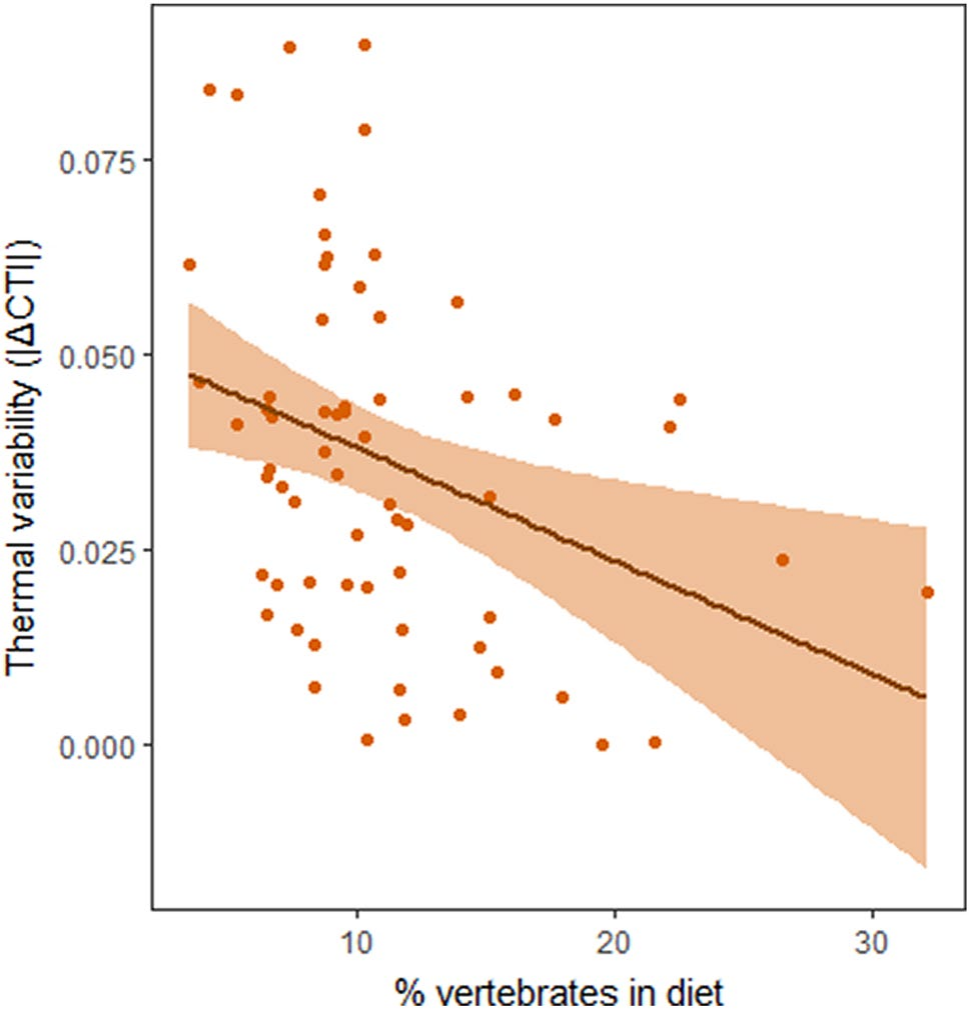
Raw diversity-thermal variability relationship of the best-ranked model in non-breeding season. Figure illustrates the raw linear relationship of the temporal change in community temperature index (|∆CTI|; thermal variability) as a function of the community-weighted mean of percentage of vertebrates in diet in the non-breeding season communities. The best-ranked diversity-thermal variability model for the breeding season communities was the null model that is not illustrated here. Grey icon indicates the season. Note that the effect of temperature trend or the spatial correlation structure are not accounted for in the model structure of the illustrated relationship (for full model statistics, see Appendix S1: Table S3). Figure was produced using R software (Version 3.5.3. URL: http://www.r-project.org/).

In accordance with our hypothesis, the higher the functional diversity measure value, the smaller the absolute change in mean CTI over time, whereby smaller CTI changes indicate lower thermal variability. In general, functional diversity, but not taxonomic or evolutionary diversity, was consistently negatively associated with thermal variability (column ‘Diversity coefficient’ in Table S3). During the non-breeding season only, the negative association between functional diversity measures and thermal variability (|∆CTI|) was statistically significant (Fig. 3, Tables S3–S4). The null model was ranked as the best model in the breeding season and the community weighted mean of the percentage of vertebrates in diet was ranked as the best model in the non-breeding season (Fig. 4, Table S3).

### Seasonal differences

The slopes of the diversity-thermal variability relationships mainly did not differ between seasons (column ‘Season interaction coefficient’ in Table S5). Moreover, the means of diversity estimates across grid cells did not largely differ between seasons for taxonomic and phylogenetic diversities (N_species_: t = -1.11, *df* = 76.18, *p* = 0.27; Phylogenetic diversity: t = − 1.71, *df* = 75.45, *p* = 0.091; means shown as vertical dashed lines in Fig. 3a,c,d,f). However, the means of diversity measures across grid cells did differ between seasons for functional diversity (Functional dispersion: t = − 3.77, *df* = 118.79, *p* < 0.001; means shown as vertical dashed lines in Fig. 3b,e). Moreover, qualitatively the variations in taxonomic and evolutionary diversity estimates were much larger in the non-breeding than in the breeding season communities, while the variation in functional diversity measure values did not differ greatly (horizontal extents of scatter points in Fig. 3).

## Discussion

In accordance with our hypotheses, we found a consistent diversity-thermal variability relationship when measuring the functional diversity of bird communities, although the relationship was not always statistically significant. The breeding season communities tended to be less thermally variable than the non-breeding season communities, but the impact of diversity on thermal variability was clearer in the non-breeding season. That is, more diverse non-breeding communities have had less change over time in their average thermal niche.

As hypothesized, functional diversity had a stronger effect on thermal variability than taxonomic or evolutionary diversity. Such an effect may stem from higher ecosystem functioning and from established species’ interactions in functionally diverse communities. Functional diversity may contribute to lower thermal variability via greater filling of the environmental niche space in the local community, leading to smaller realized niches. Therefore, functionally diverse communities could have less decrease in their functionality because they already contain several species that can occupy the vacant niche space of the declining species. Such vacant niche space could be occupied by functionally redundant species^38^ or by species that adjust or expand their realized niches (e.g., by forming stronger interactions with existing interaction partners)^39^. Birds as a taxonomic group have highly varying and complementary functional roles within communities in terms of their trophic position (e.g., herbivore, top predator) and interaction type with other species (e.g., seed dispersal mutualism, scavenger commensalism). Generally, such functional complementarity among species can stabilize communities, because it reduces competition strength via resource and enemy partitioning as well as facilitation^13^.

Community diversity can contribute to community’s climate compositional changes under climate change also via species’ interactions^40^. This was supported by our finding that thermal variability in the non-breeding season was associated with the functional diversity measure of community-weighted mean of vertebrates in diet. This measure indicates the trophic diversity and the abundance of higher trophic levels in the communities. This finding suggests that communities including top predators (and thus multiple trophic levels and long food chains) may be better able to resist climate-induced changes in the community composition^2^. In general, when the communities are less diverse, each species is likely to have fewer and stronger interactions with other species^41^. Thus, the community as a whole is less robust to short-term variations in single species’ abundances. Moreover, disturbances can affect focal species via indirect interactions^42^, such as facilitation and apparent competition^43,44^. Removal of species can therefore lead to cascading effects through the entire community^45,46^. Alternatively, the higher trophic level species, such as raptors, are also longer-lived and thus more resilient to change or persisting with extinction debt, thereby affecting the thermal variability of the bird community more strongly than short-lived species at lower trophic levels. To assess the effect of interaction network properties on community’s thermal variability under climate change at large spatial scales, systematically sampled time-series data of interactions are needed. Moreover, local studies that investigate the potential mechanisms underlying the importance of higher trophic level for thermal variability in bird communities are needed. Until such data are available, studies like ours provide the first steps towards understanding how ecological processes related to interaction networks influence community composition changes under climate change.

We did not find seasonal differences in the diversity-thermal variability relationships, but the average level of thermal variability and the variation in the diversity measures differed between seasons. That is, the mean and variation of functional diversity did not largely differ between seasons but the variation of taxonomic and evolutionary diversities did. Both the observed lower thermal variability and the smaller variation in the diversity measures of the breeding season communities may be due to different selection pressures between seasons. For example, breeding season bird communities may have an overall weaker response to climate change (i.e., fewer changes) because they are less plastic in their mobility, foraging, or other behaviours related to demographic processes^47,48^. On the other hand, birds have shown plastic behavioural changes in their breeding timing and generally advanced their breeding dates, which can compensate their need for range or abundance shifts^49^. The winter cold has so far been a more limiting factor compared to the summer heat as maximum temperatures have not yet reached upper critical temperatures for most temperate-breeding birds^50^, translating into a larger number of projected colonizations in the non-breeding season communities^51^. The northern range edges of many wintering birds tend to correlate with isotherms of average minimum temperature, likely because species reach their thermoregulatory ceiling^52,53^. However, direct climate change effects on birds, such as thermal stress, may be stronger during the breeding season when birds are less able to adjust behaviourally by moving to thermal refuges and are experiencing temperatures close to their critical maxima. Seasonal differences could also arise from differing location fidelities, as birds typically tend to return to the same breeding locations but track food resources in the non-breeding season^10,54^. Furthermore, the thermal variability was in general lower in the breeding season compared to the non-breeding season, which may reduce the statistical power to detect significant associations between diversity and thermal variability. In the future, studying the seasonal variation in diversity-thermal variability relationships is increasingly important as climate extremes and potentially also the seasonal variation are likely to increase due to climate change^20,55^.

Our results are limited to uncovering patterns of thermal variability at large spatial scale using wide and shallow analysis. To complement our results, studies with narrow and deep analysis on the mechanisms generating the observed patterns of thermal variability are needed. Thus, future studies could extend our work to study the different processes influencing diversity-thermal variability relationships at finer scales. Firstly, more detailed understanding on the thermal niches underlying thermal variability could be obtained by using not only mean thermal niches, but also the minimum, maximum and range of temperatures experienced by species within their ranges. Our study is limited to using mean temperatures only and we acknowledge that other climatic factors, such as extreme events not captured by mean temperature, can play an important role. These climatic factors should be considered in future studies performed at finer scales and aimed at unveiling the mechanisms observed in our study. Secondly, further details could be added into the thermal variability measure by assessing not only the realized thermal niches of species across their geographic range but also their physiological thermal tolerance. That is, experimental data could be useful for understanding the fundamental thermal niches of species, as species’ realized thermal niches are influenced by various other factors beyond climate, such as anthropogenic pressures, ecological interactions, and dispersal limitations^9,56^. Finally, although bird communities tend to respond mainly to temperature changes rather than precipitation changes or extreme events^57,58^, it is possible that in some areas climate change drivers beyond average temperature change are more important. For example, in drier areas in southern North America, precipitation may be a more limiting environmental determinant of species' ranges and community properties than temperature. Therefore, future studies could also assess the spatial variation in the importance of different climate change drivers on diversity-climatic niche variability relationship.

Diversity-thermal variability relationships could inform conservation decision making with knowledge on which communities are most susceptible to future climate change. When many species disappear and appear or increase and decrease within a community simultaneously, the species composition and consequently the functional composition of the community are changed. Following such compositional changes, drastic changes in ecosystem functioning may occur if a tipping point is passed^46,59,60^. Synergistic effects of climate change with other drivers may cause the communities to reach the tipping points even faster. Indeed, beyond additive effects on thermal variability, diversity can affect thermal variability of communities synergistically with anthropogenic disturbances. Anthropogenic disturbances, such as land use intensification, can cause selective extinctions of particular functional groups sensitive to land use changes^61^ and increase synchrony in population dynamics^62^. Additionally, it is important to consider that the communities that currently are resistant to the impacts of climate change may reach a tipping point where their structure rapidly changes in response to accumulated climatic debt in the future. However, concrete conservation efforts can greatly counteract negative effects of anthropogenic pressures on bird communities^63^.

Based on the results using comprehensive abundance data at a continental scale and spanning 50 years, we conclude that functionally diverse communities have fewer changes in their compositions under climate change compared to simple communities. Importantly, diversity likely has an effect on community’s overall stability through the complex interactions among species. Therefore, it is crucial to consider communities as a whole in addition to of individual species to allow forecasting climate change effects on biodiversity and ecosystem functioning. Moreover, the large-scale perspective is important because local scale processes may not always reflect continental or global patterns. Our results underscore that diversity has been able to buffer the effects of climate change on species communities, which underlines the importance of jointly considering climate and biodiversity crises.

## Material and methods

### Data

#### Bird abundances

We used bird abundance data of breeding and non-breeding seasons, collected in two monitoring schemes. For breeding season, we used data from the North American Breeding Bird Survey (BBS; http://www.pwrc.usgs.gov/), which was established in 1966 and covers over 4000 survey routes across North America^31^. Each survey route is approximately 40 km long and contains fifty stops. At each stop, the observers conduct a three-minute point count during which they record all seen or heard birds within a 400-m radius. If repeated surveys were conducted for a route in one year, we averaged the bird counts over the number of repeated surveys. For the non-breeding season, we used data from Christmas Bird Count (CBC)^64^, which was established in 1900 and includes over 2000 survey sites. The sites are circles with a diameter of 24 kms and they are surveyed on a single day between 14 December and 5 January. The observers record abundances of all birds seen or heard within the circle. In each season, we defined the local community as those bird species that were observed on a single route or site in a single year. We included data from 1966 to 2016 for both seasons. We used the same taxonomies as the original data sets. We excluded species that are considered vagrant or introduced within the study region^65^. In total, we included 586 species in the breeding season data and 698 species in the non-breeding season data (for list of included species, see Appendix S2).

To allow comparisons between breeding and non-breeding seasons within the same standardized area and to account for stochasticity due to sampling effort or other reasons among survey routes/sites, we compiled survey routes/sites into grid cells across the study area. We created grid cells of 5° × 5° and included only those grid cells that had a minimum of five unique routes or sites surveyed across all years within each season (Fig. 2). To keep the study area the same across seasons, we excluded 11 grid cells that had sufficient data for only one of the seasons (Figure S8). Thus, we totalled 65 grid cells for which data exist for both seasons (Fig. 2). To answer the study question (1) “Does community diversity affect the thermal variability of bird communities under climate change?” and (2) “Does the effect of diversity on bird communities’ thermal variability differ between breeding and non-breeding seasons?”, we compiled the seasonal datasets after averaging the within-grid cell values of thermal variability and diversity in each season.

#### Bird characteristics

As taxonomic characteristics, we obtained family information for each species following the taxonomies of the original datasets.

To quantify summary measures of functional diversity, we obtained data of eight relevant functional traits: body size (mass in g), clutch size (number of eggs), diet allocation (proportional use of food categories), diet diversity (Shannon diversity of proportional use of food categories), foraging allocation (proportional allocation of foraging time in vertical stratum categories), habitat niche breadth (number of occupied habitat categories), migratory behaviour (binary migration status), and thermal niche breadth (SD of STI)^64–70^. For more details on the trait definitions, see Appendix S1: Table S1. These traits cover major functional aspects of bird resource use and life-history^71^, which may mediate species-level responses to climate change (but see MacLean et al.^72^). Life-history traits and ecological specialization in terms of diet and habitat can influence bird species' climate change responses, for example such that the specialists' populations decline faster than those of generalists^73,74^. We standardized all continuous traits to their means and log-transformed body size, clutch size, and thermal niche breadth.

We used the phylogeny for the sets of species in breeding and non-breeding season datasets in ‘A Global Phylogeny of Birds’ (http://birdtree.org/)^75^. We obtained 100 phylogenetic trees with Ericson backbone using the full tree for the species sets. When obtaining the phylogenetic trees, we adjusted the original taxonomy of our datasets to align with that of the phylogenetic database but transformed the taxonomy back to original for later analyses.

#### Temperature

We used monthly temperature data from Earth System Research Laboratory^37^ to obtain the mean temperature for each month in each year in each grid cell. To control for potential temperature effects on community stability, we measured the temporal trend in temperature for each grid cell^10^. For the analyses of breeding season data, we calculated the annual temporal trend of average breeding season months’ temperatures (April–July), which are known to influence species’ breeding success^33,54,76^. For the analyses of non-breeding season data, we calculated the annual temporal trend of average non-breeding months’ temperatures (December-January). We used linear regressions to calculate the temporal trends in temperature (the regression slopes) within each grid cell for both seasons separately, with the mean temperature within a sampling season as the response variable and the continuous year as the explanatory variable.

### Quantification of thermal variability of bird communities

We quantified a relevant measure of community’s climate change response: thermal variability. For this measure, we first calculated the community temperature index (CTI) for each North American bird community (similarly to Lehikoinen et al.^10^) based on species temperature indices (STI;^9^). Operationally, we followed the same procedure recently adopted by Lehikoinen et al.^10^ when estimating species-specific STI values for the breeding and non-breeding seasons. Breeding season STI was calculated as the average temperature of the typical breeding months (March-August) for each species’ breeding range over the period of 1950–2000^3,9,77^. For the calculations of breeding season STIs, we used the breeding ranges provided by BirdLife International & NatureServe^65^. We restricted the area for calculating the breeding season STIs to North America (including Canada, the United States, Mexico, and Greenland) even though some species may also have populations in Central and South America. We consider this justified because individuals from South American breeding populations would never occur in the North American breeding bird surveys and many populations may be subject to different adaptations and/or selection pressures. We calculated the average temperature of the non-breeding months (December-February) over the period of 1950–2000^77^ for each species’ non-breeding range. We selected all birds that regularly overwinter in North America (i.e., the United States, Canada, and Mexico) using the distribution ranges provided by BirdLife International & NatureServe^65^. We selected the part of the distribution where the species is either resident or non-breeding. We restricted the area for calculating the non-breeding season STIs to North and South America, where the study populations are thought to spend their non-breeding months. We consider this justified because many species have wintering ranges spanning Central and South America and truncating our study region to North America when calculating non-breeding season STI values would for some species not be representative of the average non-breeding season temperature that the species experience. In general, the choice of the region used for calculating STI does not affect the subsequent results^9^, because STI is a relative measure and calculated consistently across all species within an analysis, the resulting CTI analyses are robust.

We quantified a bird community’s thermal variability as the absolute temporal trend in CTI values across years (hereafter, |∆CTI|). Within each grid cell in breeding and non-breeding seasons separately we fitted linear mixed effect models using the R package ‘lme4’^78^. CTI was the response variable, continuous year was the explanatory variable, and route or site identity was a random intercept. We estimated the thermal variability as the absolute value of the year effect slope. We considered thermal variability as a continuum of |ΔCTI| values such that the smaller values represented less thermally variable communities and the larger values represented more thermally variable communities. We also calculated the standard error of the estimated slopes to be included in the models in the later steps to propagate the uncertainty in these estimates of thermal variability.

### Quantification of diversity of bird communities

We measured the community diversity in several ways in each survey route/site in each year before averaging the information to a larger spatial scale (Figure S1). We average the diversity measures across years and survey routes/sites within grid cells because we are interested in assessing the effects of overall diversity on thermal variability in a climate change context rather than studying temporal patterns in diversity during this particular time period. To confirm the validity of this approach, we checked the temporal trends in all diversity measures and tested for correlations between averaged diversity measures across all years and diversity measures from the first decade of the study period (Figures S5 and S6, Table S2). For the first diversity measure, we quantified the taxonomic diversity as the number of species.

Secondly, as a clear consensus of the best measure for functional diversity is lacking, we quantified the functional diversity with six measures that capture different functional properties of communities. Firstly, we used: (1) functional richness^79^ and (2) functional dispersion^80^ that are based on trait distance matrices (here, Gower distances). Functional richness for multiple traits represents the amount of functional space filled by the community and is calculated as the volume of the n-dimensional convex hull. Functional dispersion represents the mean distance of individual species' trait values to the centroid of all species' (trait mean) in the community^80^. To calculate these measures, we included the above-mentioned traits and gave equal weights to each of them such that the seven categories within diet allocation received 1/7 weights and the four foraging categories received 1/4 weights. As binary occurrences and relative abundances are used for the calculation of functional richness and functional dispersion, respectively, these measures potentially capture different parts of the variation in the functional diversity of the communities. To support disentangling the potential effects of single traits on thermal variability, we quantified functional diversity using selected traits separately, similarly to Schipper et al. and Barnagaud et al.^25,81^. We calculated community weighted means of species’ (3) diet diversity, (4) thermal niche breadth, and (5) habitat niche breadth as proxies for the degree of specialization in the communities. We also calculated (6) the community weighted mean of the percentage of vertebrates in species’ diet as a proxy for the trophic diversity, that is, the presence and abundance of higher trophic levels in the community. We used the R package ‘FD’ to quantify functional diversity measures^18,80^.

Thirdly, we quantified the evolutionary diversity as Faith’s phylogenetic diversity^82^. Phylogenetic diversity can reflect the presence of unique evolutionary lineages in the local community. Faith’s phylogenetic diversity is calculated as the sum of branch lengths in the respective phylogenetic tree. We used the R package ‘Picante’^16^ to quantify phylogenetic diversity for each survey route/site using 25 randomly selected replicate trees out of the 100 obtained phylogenetic trees and averaged the 25 phylogenetic diversity values. The variation in the phylogenetic diversity values among obtained phylogenetic trees within each grid cell was relatively small in both breeding and non-breeding seasons (Figure S2).

Measures of species number, functional richness and phylogenetic diversity accounted for species’ presence-absences, while the measures of functional dispersion and community weighted means of diet diversity, thermal niche breadth, habitat niche breadth and percentage of vertebrates in diet accounted for species’ relative abundances.

### Quantification of diversity effects on thermal variability

We focused on diversity effects on thermal variability at large spatiotemporal scales and considered grid cells as the units of study, thus accounting for the potential geographic bias in the measures of thermal variability and diversity at the level of survey routes/sites. Therefore, we first calculated diversity measures for each survey route (breeding season) and site (non-breeding season) in each year. Then, we averaged their values across routes/sites within each grid cell across all years. We verified that the averaged diversity measures within grid cells across years were representative also of the diversity values of the earlier years of the dataset, by testing the correlations of the average diversity measures across years and the average diversity measures across the first ten years of data (1966–1976; Table S2).

We studied the relationships between community diversity and thermal variability using linear regressions and did not consider the temporal fluctuations in CTI, because there is often a temporal lag in the bird communities' response to climate change^10,47^ that the analyses of temporal fluctuations in CTI could not capture. These temporal lags in responses are a result of variations in species’ demography, life history, and dispersal capabilities^9,47^. We constructed linear models separately for breeding and non-breeding seasons using the R package ‘nlme’^83^. We considered thermal variability (|∆CTI|) as the response variable in all models. As explanatory variables, we included taxonomic, functional, and evolutionary diversity measures as well as the temporal temperature trend in each model. Thus, we accounted for the potential temperature effect on the diversity-thermal variability relationships. Moreover, we accounted for spatial autocorrelation by including the grid cell-specific mean spatial coordinates of the survey routes/sites, rather than coordinates of grid cell centroids, within a Gaussian correlation structure. To take account of the uncertainty potentially introduced by varying sampling efforts in |∆CTI|, we weighted the observations in the analyses with the standard errors (SE) estimated when calculating the |∆CTI| for each grid cell. Due to strong correlations among some of the diversity measures (Figure S3), we fitted several models with only single diversity measures to avoid collinearity. In addition to these models, we fitted two simpler models, with only the intercept and with only the temperature trend as explanatory variables.

We conducted a model selection procedure to assess which diversity-thermal variability model performed best in each season. We compared all models within a season using AICc designed for small sample sizes, and considered models within two AICc units to be equally supported. For model selection, we used the R package ‘MuMIn’^84^. We checked the goodness of fit of the best models by testing for the normality and variance homogeneity of the model residuals.

To test for potential differences in the diversity-thermal variability relationships between breeding and non-breeding seasons, we modelled thermal variability as a function of an interaction term of each diversity measure and season while controlling for the effect of temperature and setting the grid cell identity as a random factor (intercept). Similarly to the season-specific models, we included a Gaussian correlation structure that accounted for the grid cell-specific mean coordinates of the survey routes/sites and included standard error of CTI model estimates as model weights. For this, we compiled the grid cell-level data of breeding and non-breeding season communities.

For all data processing, analyses, and visualization, we used R software (version 3.6.3.^85^).

## Supplementary Information

Supplementary Information.

## Data Availability

The datasets used and/or analyzed during the current study available from the corresponding author on reasonable request.
