# Supplementary material for: Effects of diversity on thermal niche variation in bird communities under climate change
Source: Sci Rep. 2022 Dec 17;12:21810. doi: 10.1038/s41598-022-26248-1 (PMC9759529; doi:10.1038/s41598-022-26248-1)
Supplement: Supplementary file 1 — Supplementary Information. [file 41598_2022_26248_MOESM1_ESM.pdf]

Effects of diversity on thermal niche variation in bird communities under climate change

Emma-Liina Marjakangas, Andrea Santangeli, Alison Johnston, Nicole L. Michel, Karine Princé, Aleksi Lehikoinen

Supplementary material: Appendix S1

1 Supplementary methods

Table S1. Definitions of species' functional traits. The traits were considered either continuous, ordinal, or binary. There were no missing values for any traits.

Trait	Definition	Reference
Body size	Body mass (g). Continuous.	(Wilman et al. 2014)
Clutch size	Number of eggs laid in a single brood. Continuous.	(De Magalhães and Costa 2009, Billerman et al. 2020)
Diet allocation	Proportional use of different food categories (%). Categories: invertebrate, vertebrate, scavenge, fruit, nectar or pollen, seed, other plant material. Ordinal.	(Wilman et al. 2014)
Diet diversity	Shannon diversity of proportional (%) use of different food categories. Categories: invertebrate, vertebrate (endotherm), vertebrate (ectotherm), fish, vertebrate (unknown), scavenge, fruit, nectar or pollen, seed, other plant material. Continuous.	(Wilman et al. 2014)
Foraging allocation	Proportional allocation of foraging time to different vertical strata (%). Categories: aerial, aquatic, ground, forest. Ordinal.	(Wilman et al. 2014)
Habitat niche breadth	Number of IUCN habitat categories listed as 'Suitable'. Continuous.	(IUCN 2019)
Migratory behavior	State of being migratory. Binary.	(Billerman et al. 2020, BirdLife International 2020)
Thermal niche breadth	Standard deviation of the species temperature index (STI) across species' range. Continuous.	(BirdLife International and NatureServe 2015)

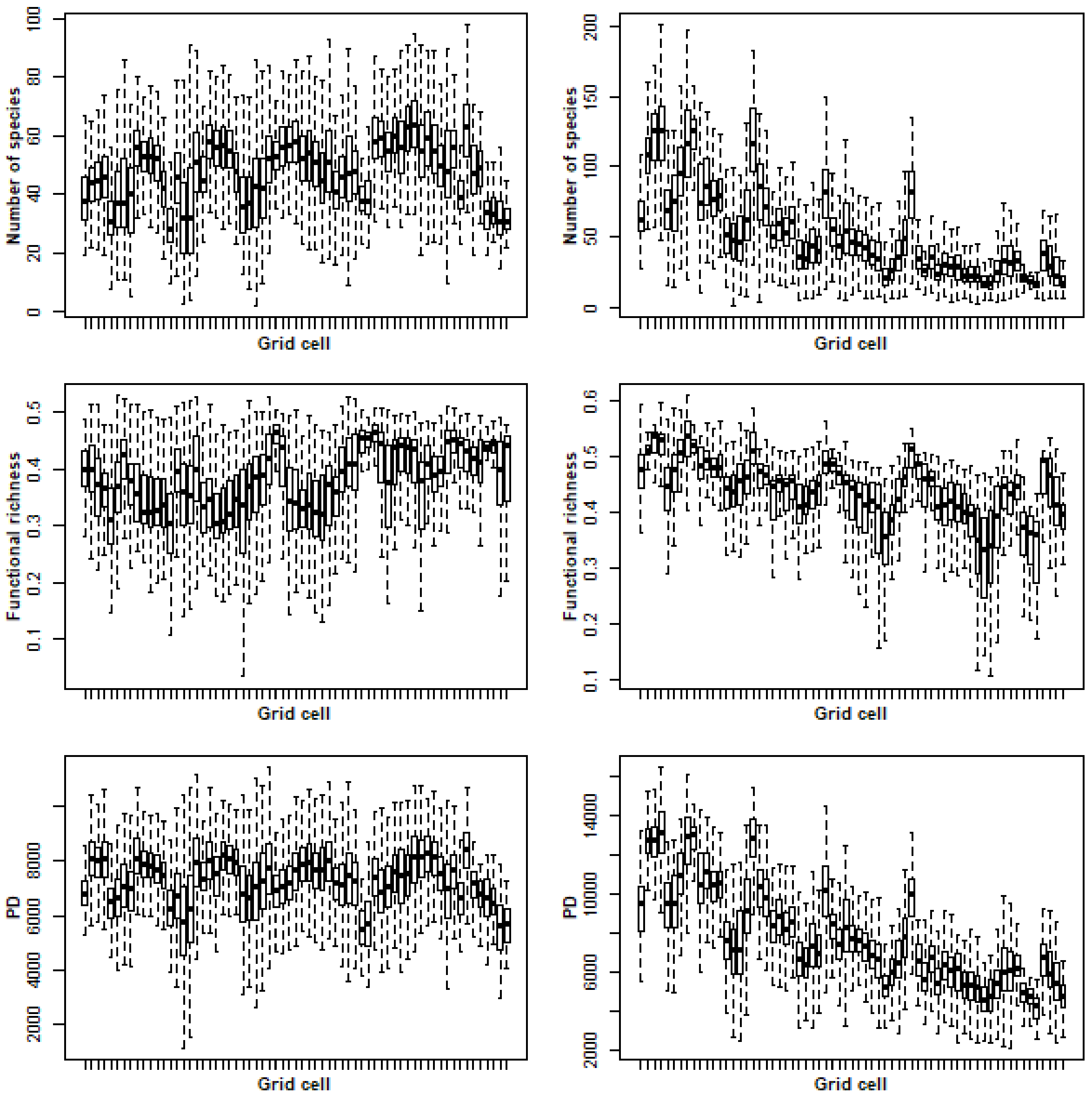

Figure S1. Variation in community diversity among survey routes/sites within grid cells (N = 65). Panels on the left represent taxonomic, functional, and evolutionary diversity measures in the breeding season. Panels on the right represent taxonomic, functional, and evolutionary diversity measures in the non-breeding season. Here, taxonomic diversity is represented as the number of species, functional diversity as functional richness, and evolutionary diversity as phylogenetic diversity (PD). For visualization, outlier values are omitted.

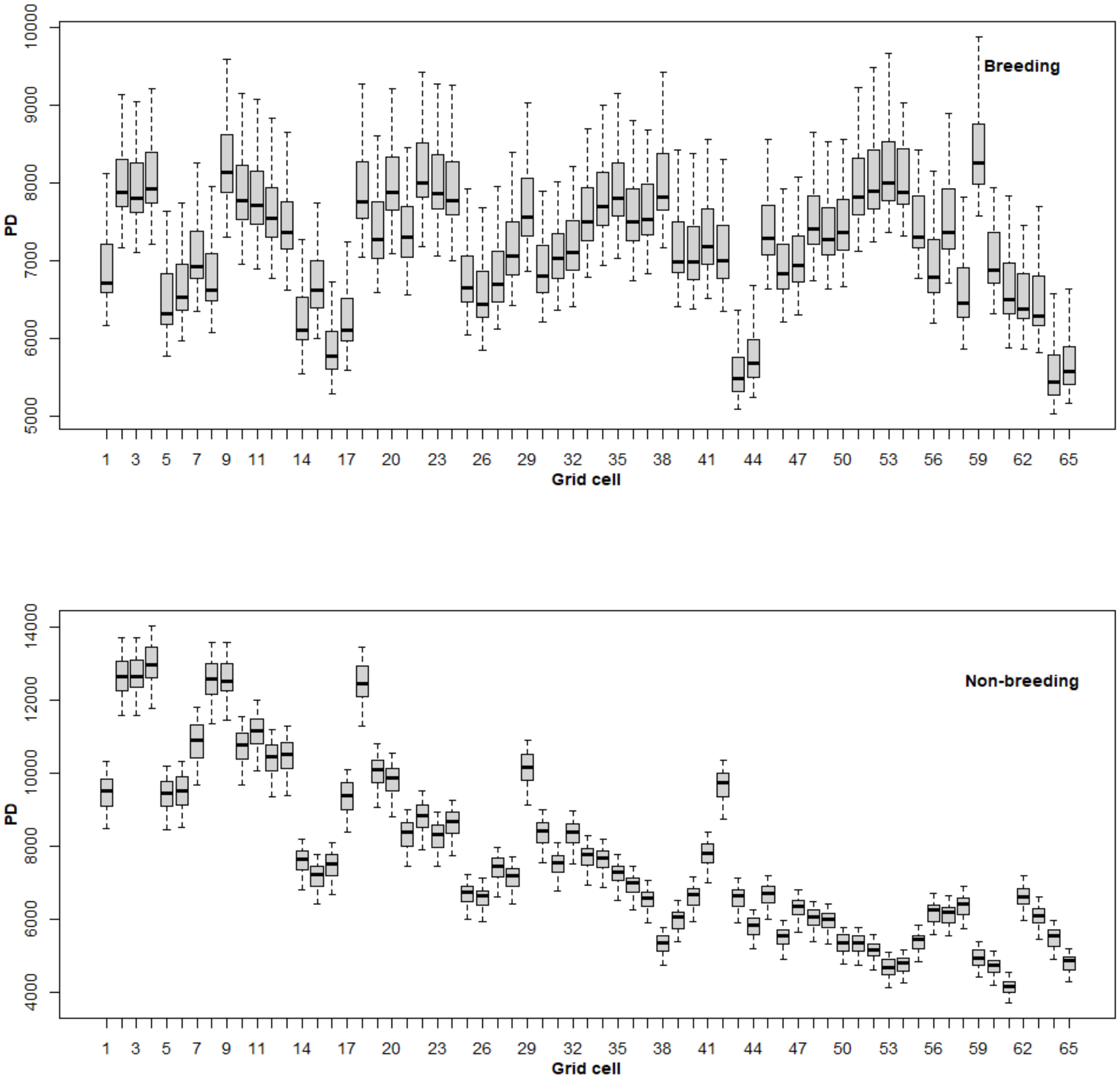

Figure S2. Variation in the mean phylogenetic diversity (PD) values in each grid cell calculated based on 25 replicated phylogenetic trees in breeding and non-breeding seasons. Phylogenetic diversity is measured as the sum of phylogenetic tree branch lengths in the phylogenetic tree of the community and then averaged over the survey routes/sites within the grid cell. Each box plot represents one grid cell (N = 65).

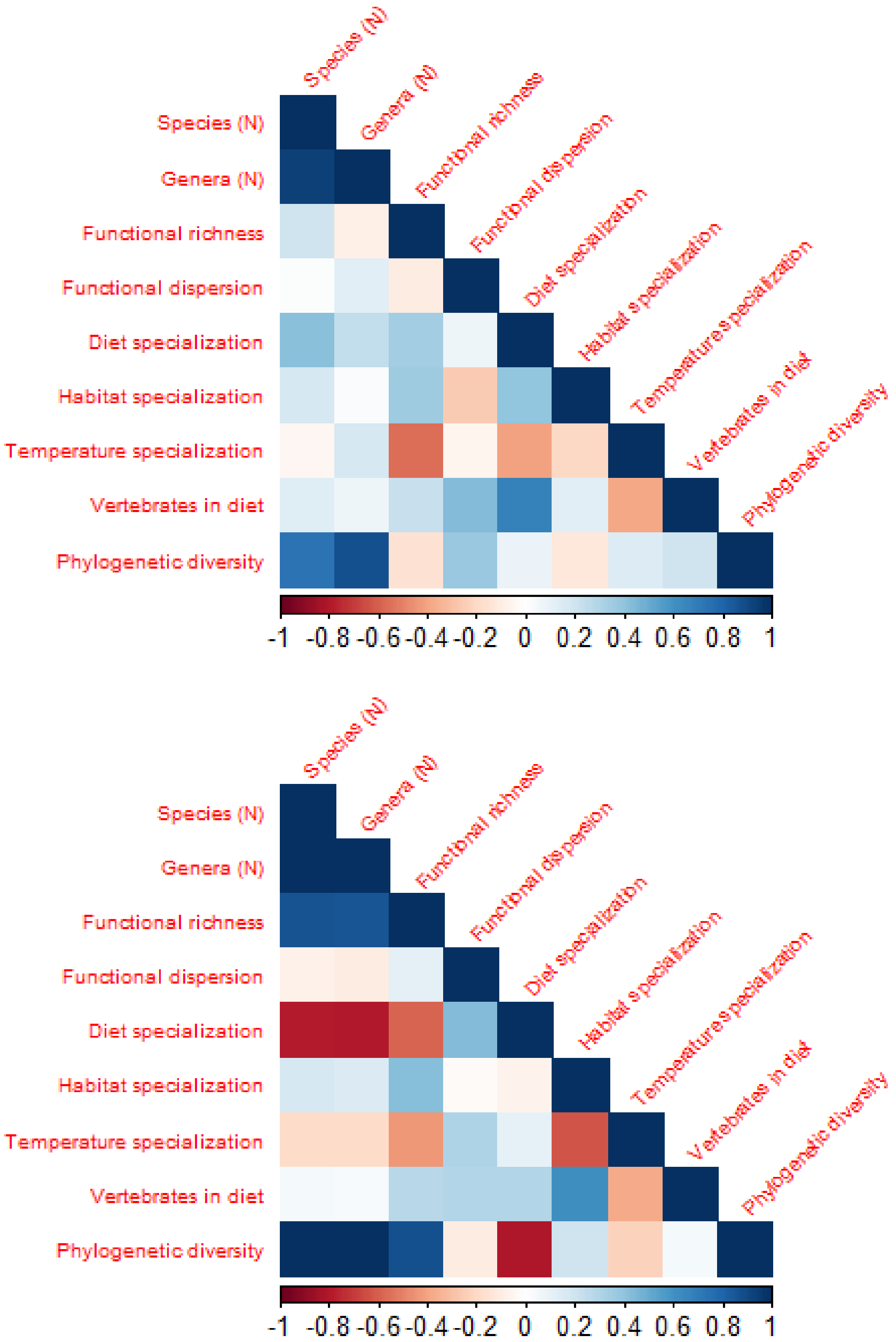

Figure S3. Pairwise correlations of taxonomic, functional, and evolutionary diversity measures quantified with Pearson's correlation coefficients. The upper and lower panels describe the diversity measure correlations within grid cells for breeding and non-breeding season data, respectively. Diet specialization = Community weighted mean of diet diversity, Habitat specialization = Community weighted mean of habitat niche breadth, Temperature specialization = Community weighted mean of thermal niche breadth, Vertebrates in diet = Community weighted mean of vertebrates in diet. For description of diversity measures, see 'Methods' in the main text.

Table S2. Correlations between community diversity measures in the first decade of the dataset (1966-1976) and in the full dataset (1966-2016) in breeding and non-breeding seasons.

	Correlation coefficient	
**Diversity measure**	**Breeding season**	**Non-breeding season**
Number of species	0.94	0.99
Functional richness	0.87	0.92
Functional dispersion	0.94	0.66
Community weighted mean of diet diversity	0.95	0.87
Community weighted mean of thermal niche breadth	0.79	0.94
Community weighted mean of habitat niche breadth	0.94	0.88
Community weighted mean of percentage of vertebrates in diet	0.87	0.89
Phylogenetic diversity	0.92	0.99

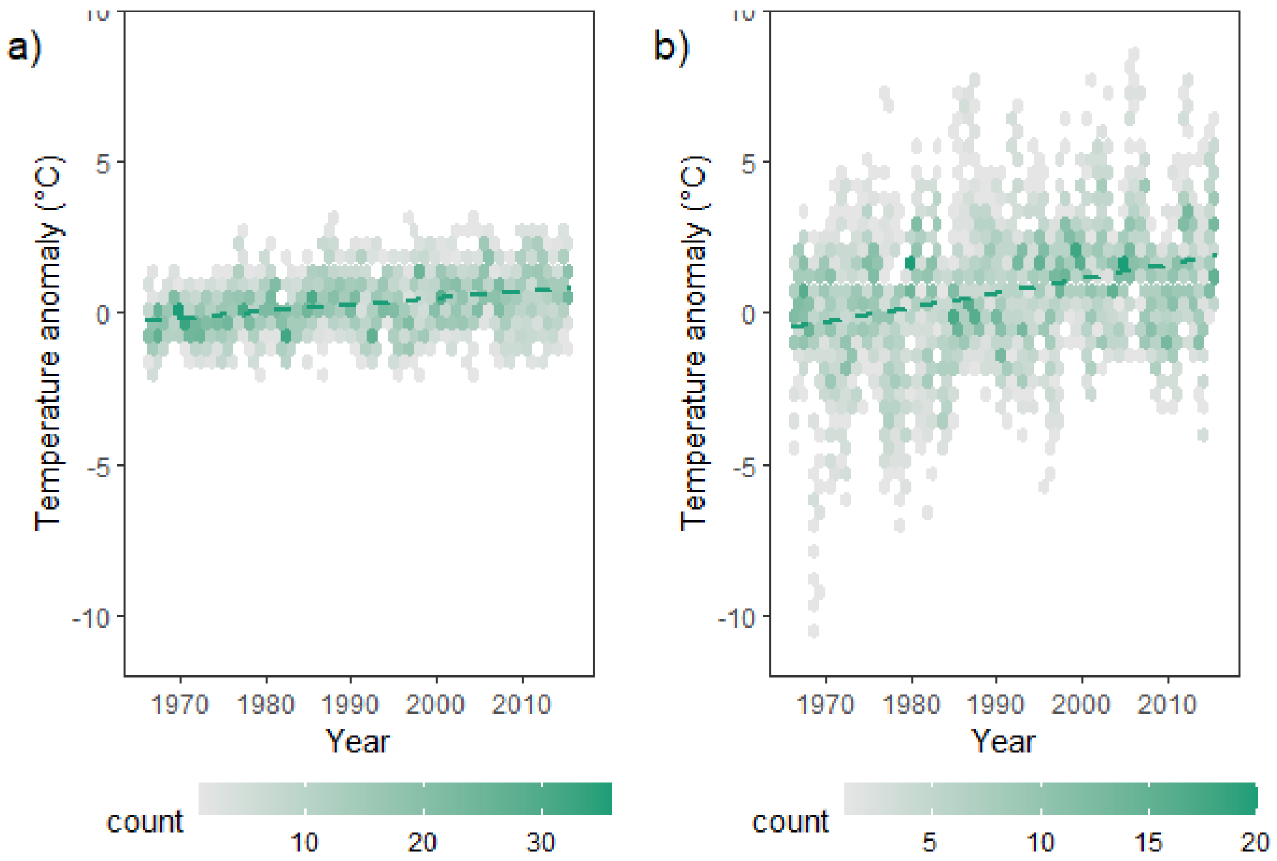

Figure S4. Temporal trends in climate across the study area between 1966 and 2016. As a proxy for measuring regional climate change, we used the observed monthly temperature anomalies in 5° × 5° grid cell data from Earth System Research Laboratory (Jones et al., 2012). Panels a) and b) show the linear temperature trend in the breeding and non-breeding season data, respectively. In both panels, hexagon color gradient indicates the number of observations (here, sampling routes/sites in a year) and the dashed line indicates the linear fit of the regression model: temperature anomaly ~ year.

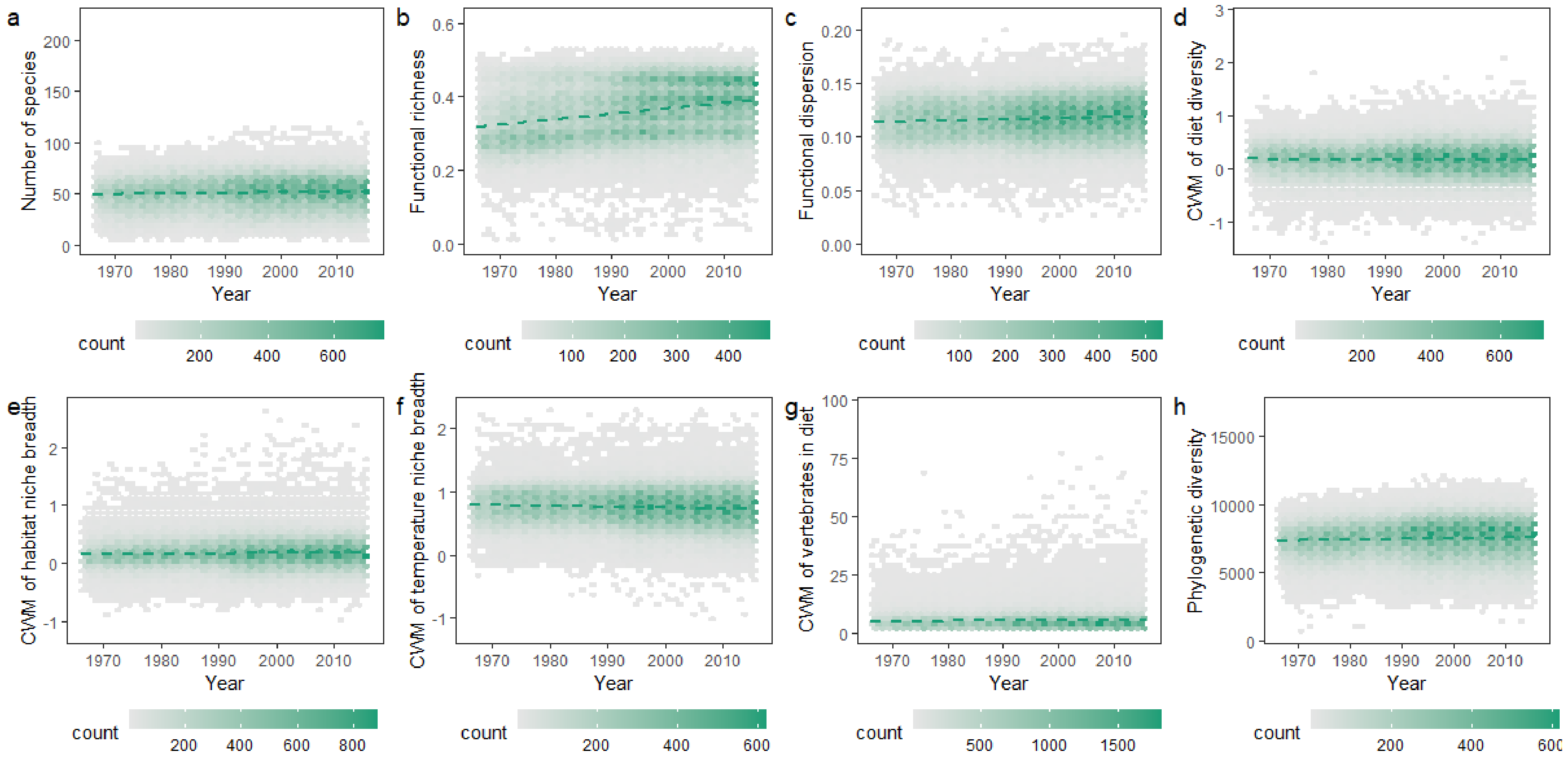

Figure S5. Temporal variation in diversity measures in the breeding season bird communities. Each panel shows the temporal variation in one diversity measure (indicated on the y-axis). In all panels, hexagon color gradient indicates the number of observations (here, sampling routes in a year) and the dashed line indicates the linear fit of the regression model: diversity measure ~ year. In panels d-g, CWM stands for community-weighted mean of the particular trait.

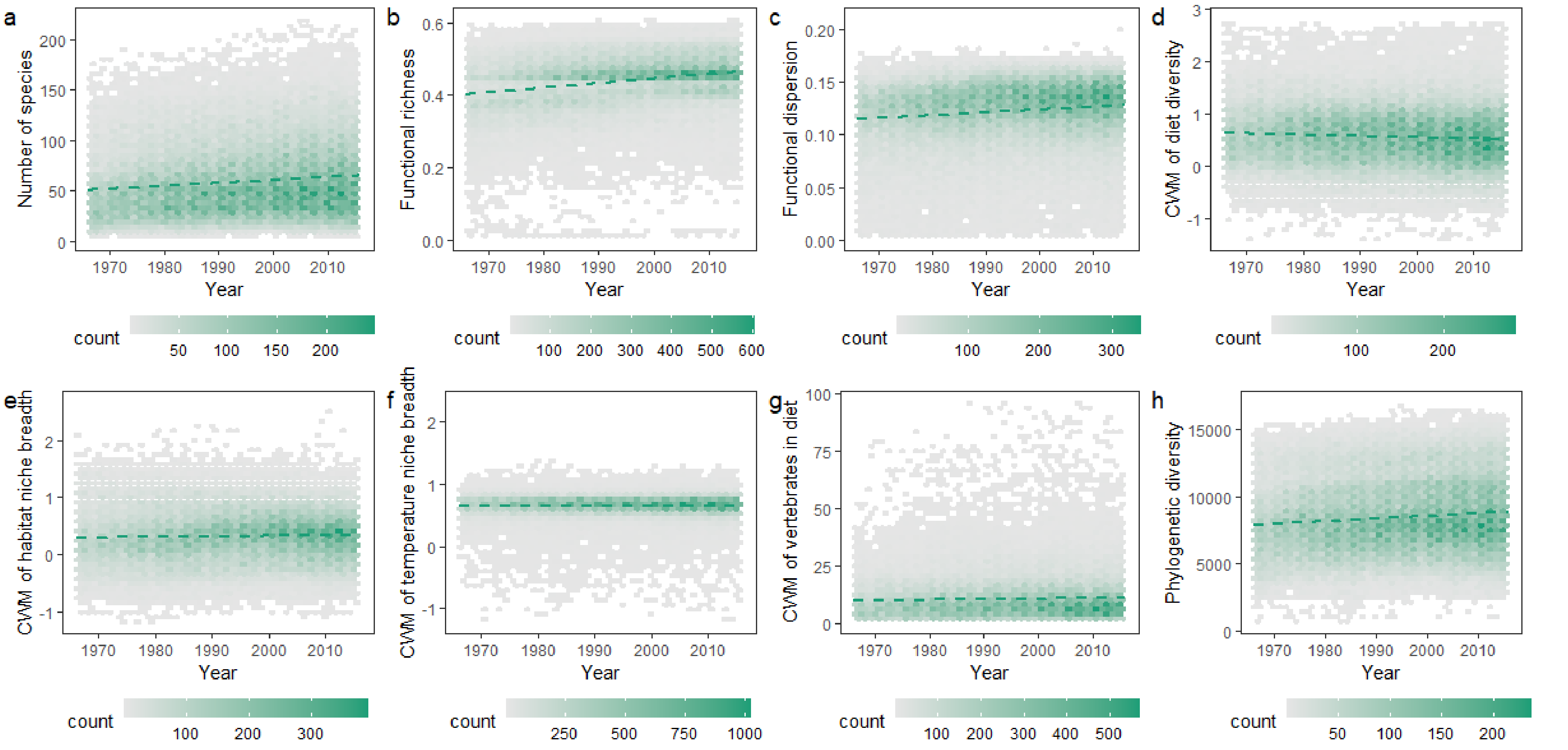

Figure S6. Temporal variation in diversity measures in the non-breeding season bird communities. Each panel shows the temporal variation in one diversity measure (indicated on the y-axis). In all panels, hexagon color gradient indicates the number of observations (here, sampling sites in a year) and the dashed line indicates the linear fit of the regression model: diversity measure ~ year.

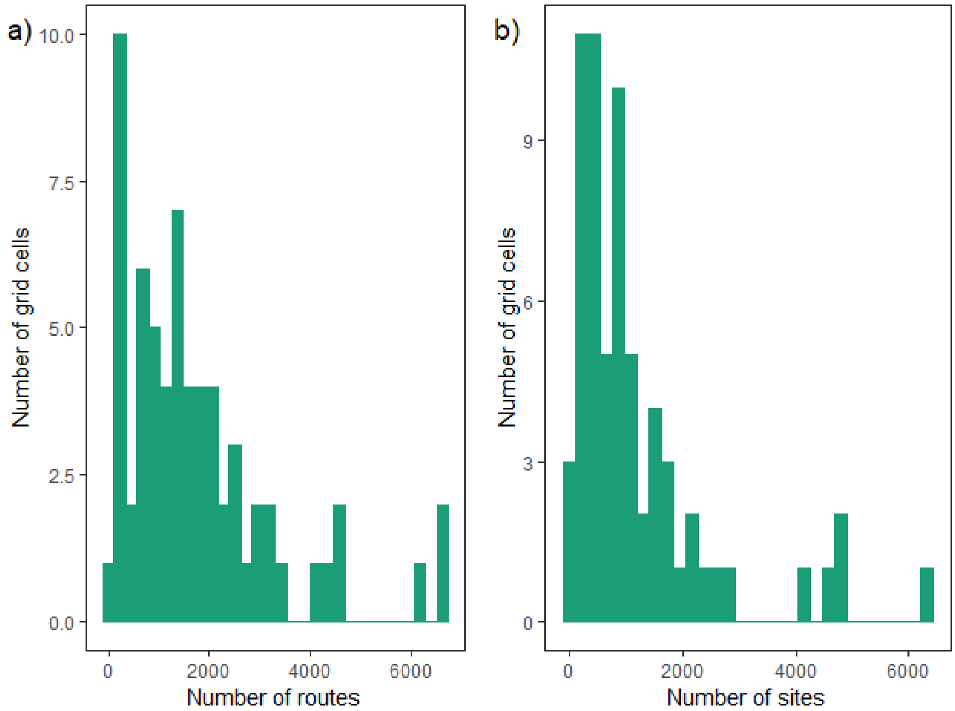

Figure S7. Variation in the number of sampling routes/sites within grid cells across years in the breeding (panel a) and non-breeding (panel b) season data.

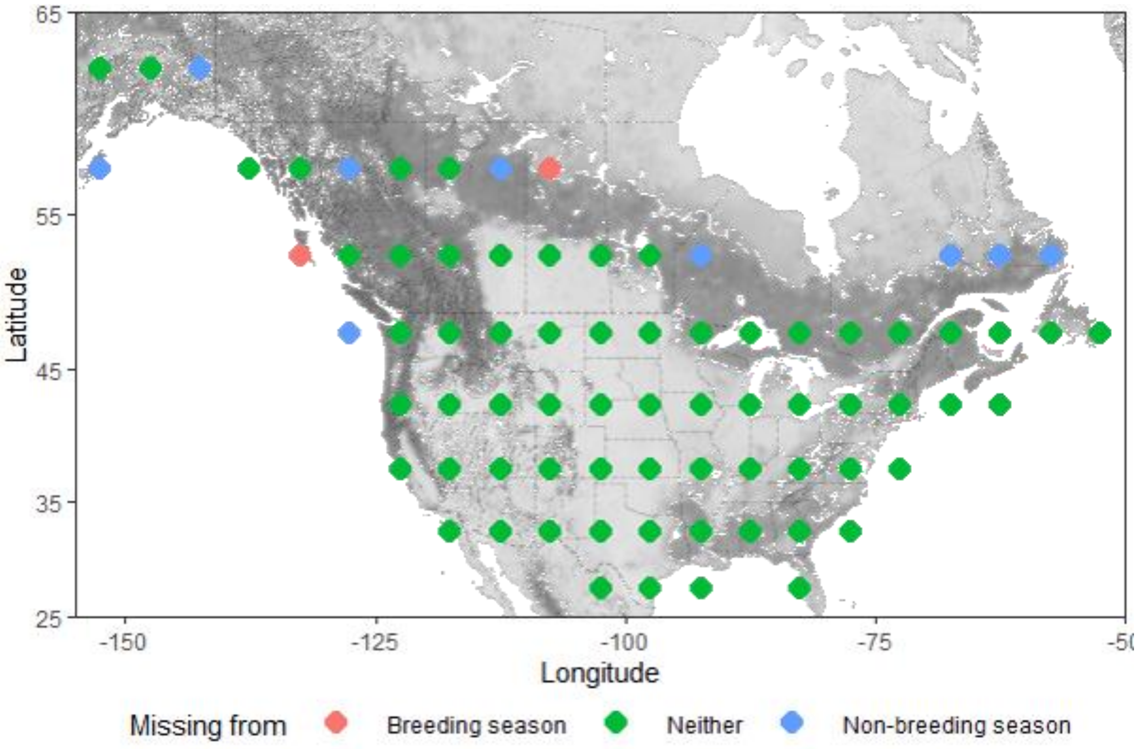

Figure S8. Spatial locations of 5 x 5 degree grid cells across North America. The red and blue circles show the distribution of excluded grid cells that included data only from one of the seasons. Red circles illustrate the grid cells that had data of the non-breeding season bird communities, but not of the breeding season bird communities. On the contrary, blue circles illustrate the grid cells

that had data of the breeding season bird communities, but not of the non-breeding season bird communities.

2 Supplementary results

Among all the diversity-thermal variability models (response variable: |ΔCTI|), models including community diversity measures performed better than the null model in the non-breeding but not in the breeding season (Table S3). In the breeding season, the null model performed best, while the second best model included the functional dispersion. In the non-breeding season, the diversity-thermal variability models that performed the best included the community weighted mean of vertebrates in diet, while the second best model included the community weighted mean of habitat niche breadth. In the non-breeding season, |ΔCTI| was smaller when diversity was higher (Table S4).

We mainly did not find differences in the diversity-thermal variability relationships between breeding and non-breeding seasons (Table S5). More specifically, the interaction term was not significant in any of the models, except in the case of functional richness. The relationship between thermal variability and functional richness was stronger in the non-breeding season.

We inspected visually the best models for each season and found the model residuals normal and variances homogenetic in case of the best model fitted to non-breeding season data (Figures S4-S5).

Table S3. Output of linear models on the diversity-thermal variability relationships (response variable: |ΔCTI|) between 1966 and 2016. Each row represents one hypothesis and one linear model with the same response variable and different explanatory variables. All models include a Gaussian spatial correlation structure and are weighted by the grid cell-specific standard errors obtained from the models CTI ~ Year. The best model(s) were identified using Akaike Information Criteria for small samples (AICc). The rows for each season are ordered from best to worst model according to ΔAICc, starting with the best supported model (ΔAICc=0). We identified as the best model(s) those models for which ΔAICc ≤ 2. Pseudo R^2 for gls-models are represented to indicate models' explanatory power. The coefficient and standard error of diversity measure variables in each model are shown in separate columns. Similarly, the coefficient and p-value of temperature change variable in each model are shown in separate columns.

Hypothesis	Model	ΔAICc	Pseudo R²	Diversity coef	Diversity SE	ΔTemp coef	ΔTemp p-value
**Breeding season**							
Null	|ΔCTI| ~ 1	0.000	*	*	*	*	*
Temperature	|ΔCTI| ~ ΔTemperature	2.145	0.004	*	*	0.0512	0.7238
Functional	|ΔCTI| ~ ΔTemperature + Functional dispersion	2.972	0.014	-0.130	0.107	0.0666	0.6455
Taxonomic	|ΔCTI| ~ ΔTemperature + Number of species	3.556	0.009	< 0.001	< 0.001	0.1077	0.4934
Functional	|ΔCTI| ~ ΔTemperature + Community weighted mean of habitat niche breadth	3.777	0.001	-0.008	0.009	0.0157	0.9176
Functional	|ΔCTI| ~ ΔTemperature + Community weighted mean of thermal niche breadth	4.088	0.027	-0.004	0.007	0.0376	0.7978

Evolutionary	|ΔCTI| ~ ΔTemperature + Phylogenetic diversity	4.360	0.008	< 0.001	< 0.001	0.027	0.8666
Functional	|ΔCTI| ~ ΔTemperature + Community weighted mean of diet diversity	4.477	0.001	-0.001	0.007	0.0503	0.7311
Functional	|ΔCTI| ~ ΔTemperature + Community weighted mean of vertebrates in diet	4.487	0.002	< 0.001	0.001	0.0505	0.7292
Functional	|ΔCTI| ~ ΔTemperature + Functional richness	15.937	0.004	-0.034	0.028	0.048	0.7462
**Non-breeding season**							
Functional	|ΔCTI| ~ ΔTemperature + Community weighted mean of vertebrates in diet	0.000	0.110	-0.002	0.001	-0.0775	0.4757
Functional	|ΔCTI| ~ ΔTemperature + Community weighted mean of habitat niche breadth	3.494	0.048	-0.034	0.010	-0.2059	0.1014
Null	|ΔCTI| ~ 1	9.055	*	*	*	*	*
Functional	|ΔCTI| ~ ΔTemperature + Community weighted mean of diet diversity	9.668	0.001	-0.023	0.011	0.1035	0.458
Temperature	|ΔCTI| ~ ΔTemperature	11.207	< 0.001	*	*	-0.0401	-0.0401
Functional	|ΔCTI| ~ ΔTemperature + Community weighted mean of thermal niche breadth	12.317	0.002	0.024	0.021	-0.1233	0.3804
Evolutionary	|ΔCTI| ~ ΔTemperature + Phylogenetic diversity	13.210	0.051	< 0.001	< 0.001	-0.1041	0.5175
Taxonomic	|ΔCTI| ~ ΔTemperature + Number of species	13.211	0.050	< 0.001	< 0.001	-0.0951	0.5279
Functional	|ΔCTI| ~ ΔTemperature + Functional dispersion	13.302	0.011	-0.176	0.354	-0.0346	0.77
Functional	|ΔCTI| ~ ΔTemperature + Functional richness	35.366	0.086	-0.145	0.110	-0.3093	0.1285

* Not relevant for the model

Table S4. Linear model outputs for the best diversity-thermal variability relationships (response variable: |ΔCTI|) in the breeding (B) and non-breeding (NB) seasons (for all models, see Table S3). All models include a Gaussian spatial correlation structure and are weighted by the grid cell-specific standard errors obtained from the models CTI ~ Year.

	**Model**	
**Season**	Breeding	Non-breeding
**Model structure**	|ΔCTI| ~ 1	|ΔCTI| ~ ΔTemperature + Community weighted mean of vertebrates in diet
**ΔAICc**	0	0
**Model pseudo R^2**	*	0.110
**df**	64	62
**Temperature coefficient**	*	-0.078
**Temperature p**	*	0.476
**Diversity measure coefficient**	*	-0.002
**Diversity measure p**	*	<0.001

* Not relevant for the model

Table S5. Magnitude and significance of the seasonal effect on the diversity-thermal variability relationships (response variable: |ΔCTI|) between 1966 and 2016. The coefficient and p-value of the interaction term for season and diversity measure are given in the right-most columns. All models include a Gaussian spatial correlation structure and are weighted by the grid cell-specific standard errors obtained from the models CTI ~ Year. Breeding season was set as the baseline.

Hypothesis	Model	Season interaction coefficient	Season interaction p
Taxonomic	ΔCTI ~ ΔTemperature + Number of species * Season + (1|Grid cell)	<0.001	0.616
Functional	ΔCTI ~ ΔTemperature + Community weighted mean of vertebrates in diet * Season + (1|Grid cell)	<0.001	0.760
Functional	ΔCTI ~ ΔTemperature + Community weighted mean of habitat niche breadth * Season + (1|Grid cell)	-0.008	0.693
Functional	ΔCTI ~ ΔTemperature + Functional dispersion * Season + (1|Grid cell)	-0.300	0.405
Functional	ΔCTI ~ ΔTemperature + Community weighted mean of diet diversity * Season + (1|Grid cell)	0.009	0.564
Functional	ΔCTI ~ ΔTemperature + Community weighted mean of thermal niche breadth * Season + (1|Grid cell)	0.001	0.967
Functional	ΔCTI ~ ΔTemperature + Functional richness * Season + (1|Grid cell)	-0.170	0.046
Evolutionary	ΔCTI ~ ΔTemperature + Phylogenetic diversity * Season + (1|Grid cell)	<0.001	0.963

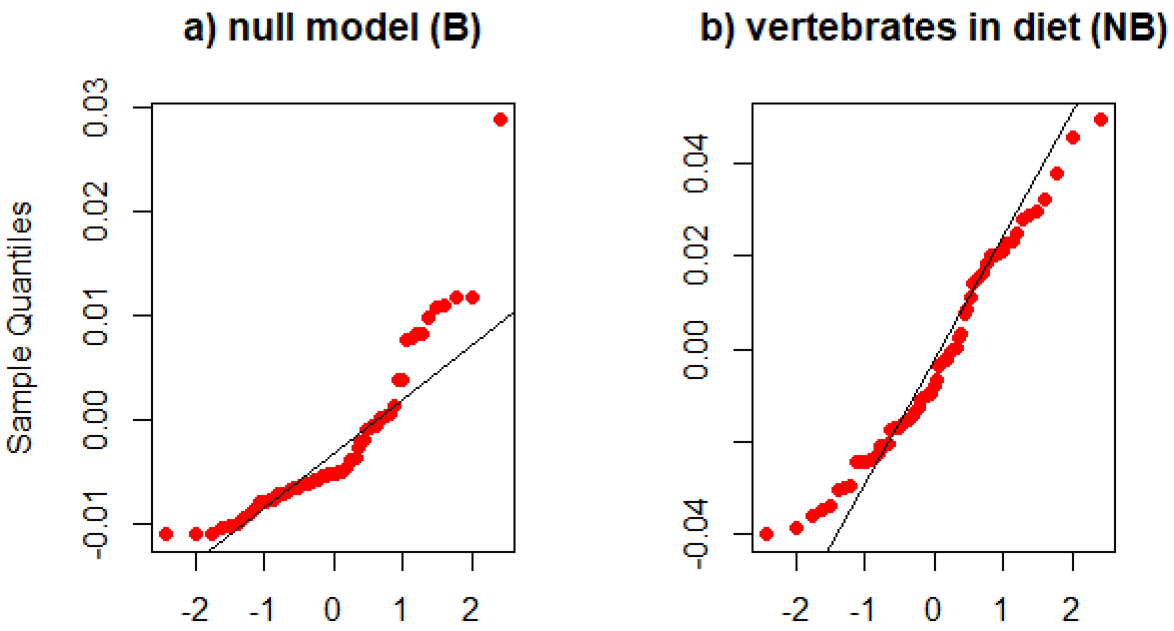

Figure S9. Normality of residuals in the best models (response variable: |ΔCTI|) in the breeding (B) and non-breeding seasons (NB) (for the ranking of all models, see Table S3). Each panel represents one of the models listed in Table S4.

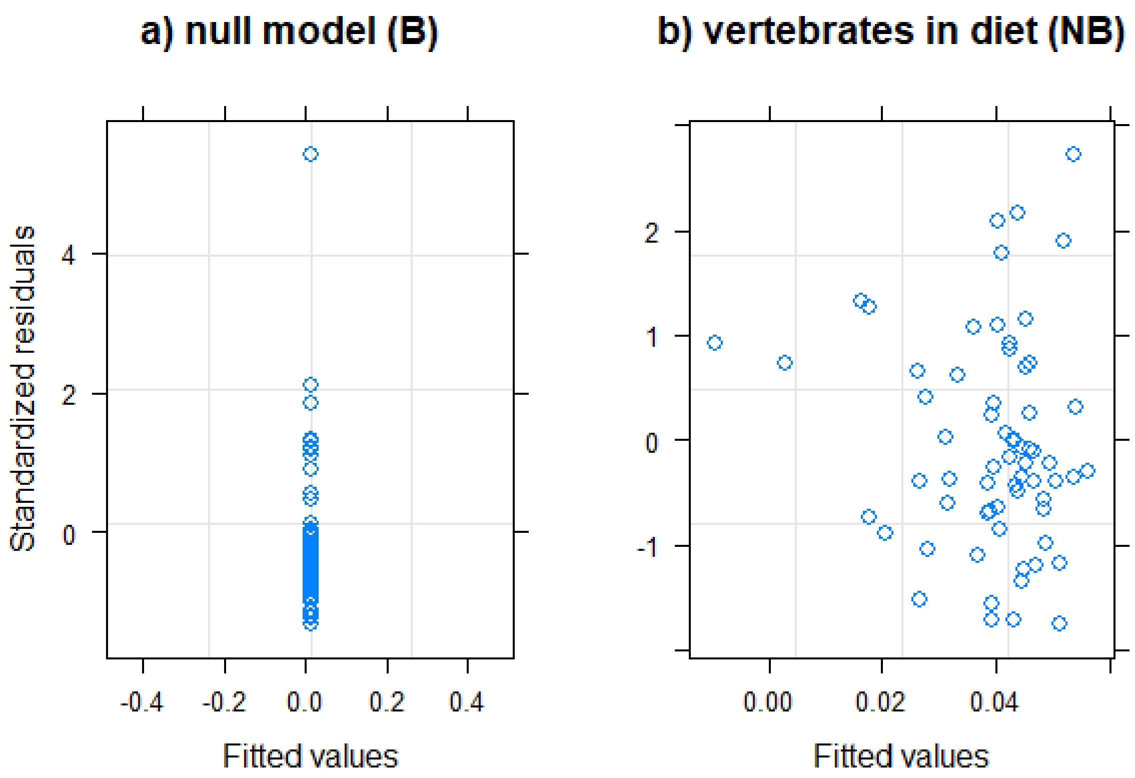

Figure S10. Variance homogeneity of residuals of the best models (response variable: |ΔCTI|) in the breeding (B) and non-breeding (NB) seasons (for the ranking of all models, see Table S3). Each panel represents one of the models listed in Table S4.

2.1 Sensitivity analyses

To account for the potential variation in temporal trend strengths of CTI and in the development of data collection methods, we conducted a sensitivity analysis of thermal variability models using subsets of the full datasets. By 1980, use of optical tools for bird observations became a standard, potentially increasing the detection probabilities.

We refitted the diversity-thermal variability models using thermal variability measure and community data between years 1980 and 2016. We found that |ΔCTI| did not differ between the full and subset data analyses, such that the means across the grid cells in the breeding season were 0.009 and 0.009 and in the non-breeding season were 0.036 and 0.036, in the full and subset data analyses respectively. Qualitatively the general diversity-thermal variability results remained parallel in the sensitivity analysis as in the full data analysis (compare Figures S11 and Figure 4 in the main text). However, the ranking of the models differed, such that in the breeding season, the temperature model was ranked the best and in the non-breeding season, the evolutionary and taxonomic diversity models were ranked above other models (Table S9). However, the functional richness model in the non-breeding season had the steepest diversity-thermal variability slope but was not top-ranked due to high variation (Table S9).

We also tested for the relationship between temperature change and thermal variability in each season and found no significant relationships (Figure S12).

Table S9. Output of linear models on the diversity-thermal variability relationships (response variable: |ΔCTI|) for data between years 1980 and 2016. Each row represents one hypothesis and one linear model with the same response variable and different explanatory variables. All models include a Gaussian spatial correlation structure and are weighted by the grid cell-specific standard errors obtained from the models CTI ~ Year. The best model(s) were identified using Akaike Information Criteria for small samples. The rows for each season are ordered from best to worst model according to ΔAICc, starting with the best supported model (ΔAICc=0). We identified as the best model(s) those models for which ΔAICc ≤ 2. Pseudo R^2 for gls-models are represented to indicate models' explanatory power. The coefficient and standard error of diversity measure variables in each model are shown in separate columns.

Hypothesis	Model	ΔAICc	Pseudo R²	Diversity coefficient	Diversity SE
**Breeding season**					
Temperature	|ΔCTI| ~ ΔTemperature	0.000	0.045	*	*
Functional	|ΔCTI| ~ ΔTemperature + Community weighted mean of diet diversity	0.873	0.061	0.008	0.006
Functional	|ΔCTI| ~ ΔTemperature + Community weighted mean of vertebrates in diet	1.670	0.061	<0.001	0.001
Functional	|ΔCTI| ~ ΔTemperature + Community weighted mean of thermal niche breadth	1.829	0.055	-0.005	0.007
Evolutionary	|ΔCTI| ~ ΔTemperature + Phylogenetic diversity	2.019	0.046	<0.001	<0.001
Taxonomic	|ΔCTI| ~ ΔTemperature + Number of species	2.242	0.045	<0.001	<0.001
Functional	|ΔCTI| ~ ΔTemperature + Community weighted mean of habitat niche breadth	2.338	0.045	0.001	0.009
Functional	|ΔCTI| ~ ΔTemperature + Functional dispersion	2.346	0.044	-0.012	0.113
Functional	|ΔCTI| ~ ΔTemperature + Functional richness	7.931	0.025	0.030	0.031
Null	|ΔCTI| ~ 1	11.562	*	*	*
**Non-breeding season**					
Evolutionary	|ΔCTI| ~ ΔTemperature + Phylogenetic diversity	0.000	0.081	<0.001	<0.001
Taxonomic	|ΔCTI| ~ ΔTemperature + Number of species	0.985	0.066	<0.001	<0.001
Null	|ΔCTI| ~ 1	2.678	*	*	*
Temperature	|ΔCTI| ~ ΔTemperature	4.278	0.002	*	*
Functional	|ΔCTI| ~ ΔTemperature + Community weighted mean of vertebrates in diet	4.460	0.054	-0.001	0.001
Functional	|ΔCTI| ~ ΔTemperature + Community weighted mean of habitat niche breadth	6.237	0.011	-0.007	0.011
Functional	|ΔCTI| ~ ΔTemperature + Community weighted mean of diet diversity	6.542	0.002	0.003	0.010
Functional	|ΔCTI| ~ ΔTemperature + Functional dispersion	6.604	0.003	-0.066	0.350
Functional	|ΔCTI| ~ ΔTemperature + Community weighted mean of thermal niche breadth	6.618	0.002	0.003	0.023
Functional	|ΔCTI| ~ ΔTemperature + Functional richness	16.548	0.301	-0.131	0.064

* Not relevant for the model

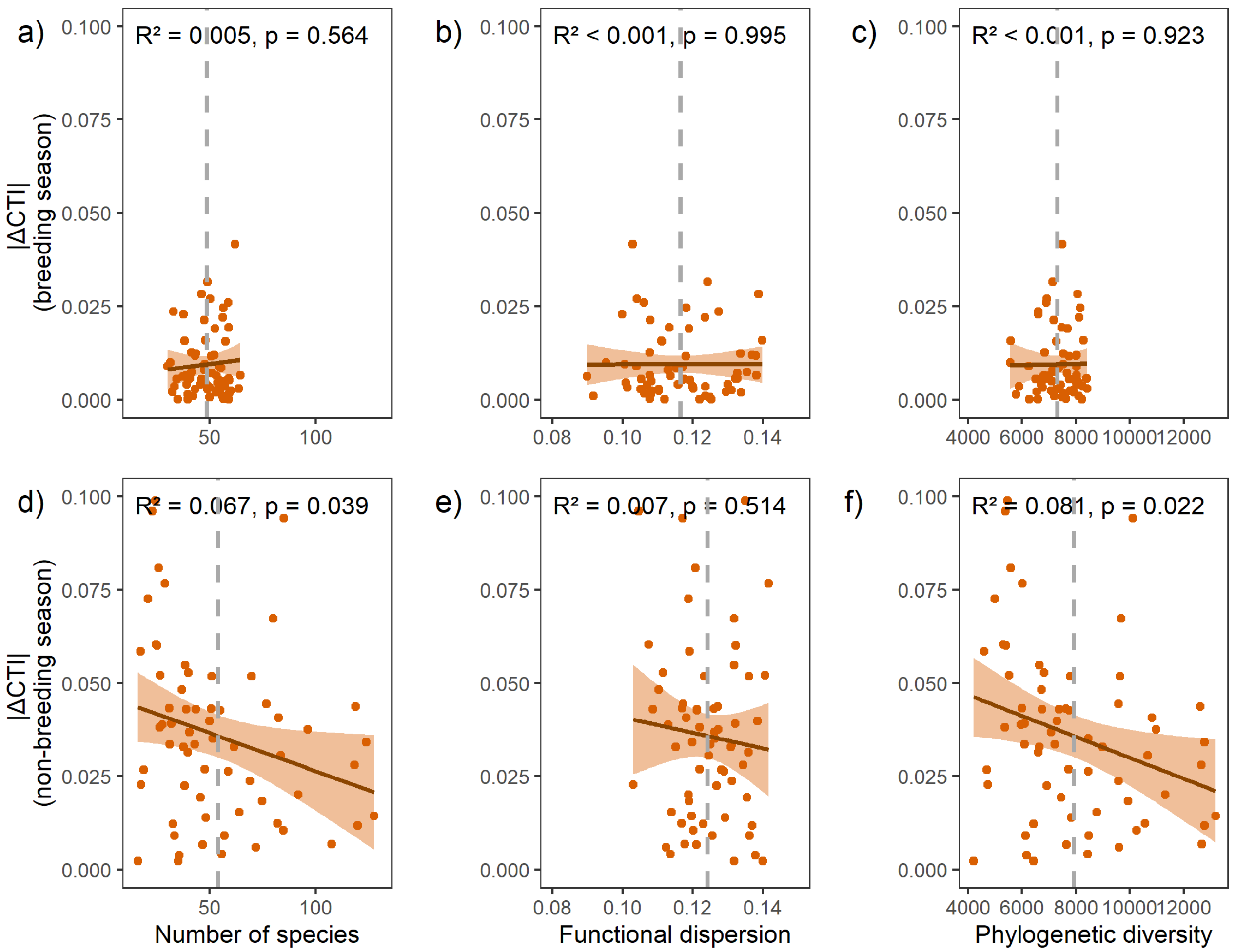

Figure S11. Examples of diversity-thermal variability relationships (response variable: |ΔCTI|) in a)-c) breeding and d)-f) non-breeding seasons for data between years 1980 and 2016. The linear relationships with metrics of taxonomic, functional and evolutionary diversity for each season are illustrated. Thereby, panels a) and d) illustrate the temporal change in community temperature index (|ΔCTI|) as a function of the number of species, panels b) and e) illustrate |ΔCTI| as a function of the functional dispersion, and panels c) and f) illustrate |ΔCTI| as a function of the phylogenetic diversity within the community. The model explanatory power and statistical significance are labelled at the top of each panel. Note that the effect of temperature trend is not accounted for in the model structure of the illustrated relationships (see Table S7).

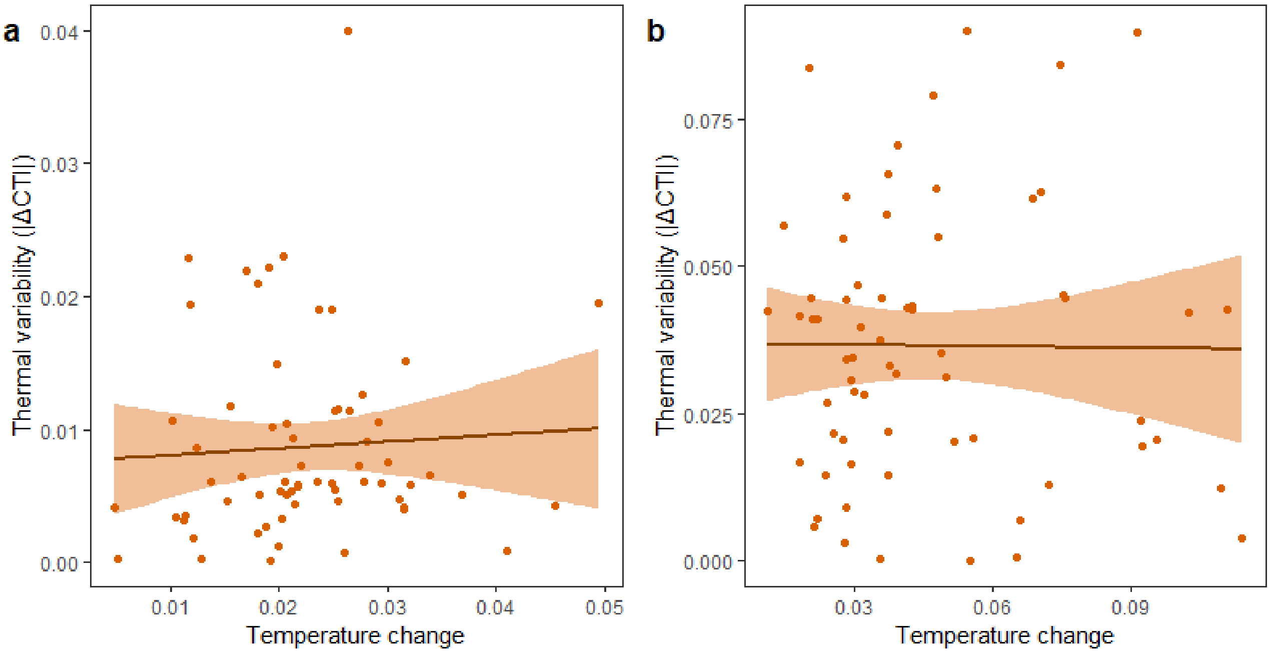

Figure S12. Thermal variability ~ temperature change relationships for the breeding (panel a) and the non-breeding (panel b) season bird communities. Each orange circle represents one grid cell. The solid lines represent the linear relationships. Neither of the relationships was statistically significant (breeding season: temperature coefficient = 0.051, SE = 0.106, p = 0.633; non-breeding season: temperature coefficient -0.008, SE = 0.110, p = 0.941). This means that the thermal variability was neither higher nor lower in areas where climate (temperature) change has been more intense.

3 Supplementary references

Billerman, S. M. et al. 2020. Birds of the World. in press.

BirdLife International 2020. IUCN Red List for birds.

BirdLife International and NatureServe 2015. Bird species distribution maps of the world. Version 4.0. in press.

De Magalhães, J. P. and Costa, J. 2009. A database of vertebrate longevity records and their relation to other life-history traits. - J. Evol. Biol. 22: 1770–1774.

IUCN 2019. The IUCN Red List of Threatened Species. Version 2019-2.

Wilman, H. et al. 2014. EltonTraits 1.0: Species-level foraging attributes of the world’s birds and mammals. - Ecology 95: 2027–2027.

Supplementary material: Appendix S2

1 List of species included in the datasets

**Species in breeding season data**	**Species in non-breeding season data**
Acanthis flammea	*Acanthis flammea*
Accipiter cooperii	*Accipiter cooperii*
Accipiter gentilis	*Accipiter gentilis*
Accipiter striatus	*Accipiter striatus*
Actitis macularius	*Actitis macularius*
Aechmophorus clarkii	*Aechmophorus clarkii*
Aechmophorus occidentalis	*Aechmophorus occidentalis*
Aegolius acadicus	*Aegolius acadicus*
Aegolius funereus	*Aegolius funereus*
Aeronautes saxatalis	*Aeronautes saxatalis*
Agelaius phoeniceus	*Aethia cristatella*
Agelaius tricolor	*Agelaius phoeniceus*
Aimophila ruficeps	*Agelaius tricolor*
Aix sponsa	*Aimophila ruficeps*
Alca torda	*Aix sponsa*
Amazilia violiceps	*Alca torda*
Amazilia yucatanensis	*Alle alle*
Amazona viridigenalis	*Amazilia beryllina*
Ammodramus savannarum	*Amazilia violiceps*
Ammospiza caudacuta	*Amazilia yucatanensis*
Ammospiza leconteii	*Amazona aestiva*
Ammospiza maritima	*Amazona albifrons*
Ammospiza nelsoni	*Amazona amazonica*
Amphispiza bilineata	*Amazona auropalliata*
Anas acuta	*Amazona autumnalis*
Anas crecca	*Amazona farinosa*
Anas fulvigula	*Amazona finschi*
Anas platyrhynchos	*Amazona ochrocephala*
Anas rubripes	*Amazona oratrix*
Anhinga anhinga	*Amazona viridigenalis*
Anser albifrons	*Ammodramus savannarum*
Anser caerulescens	*Amphispiza bilineata*
Anthus rubescens	*Amphispiza quinquestriata*
Anthus spragueii	*Anas acuta*
Antigone canadensis	*Anas americana*
Antrostomus arizonae	*Anas bahamensis*
Antrostomus carolinensis	*Anas clypeata*
Antrostomus vociferus	*Anas crecca*
Aphelocoma californica	*Anas cyanoptera*
Aphelocoma coerulescens	*Anas discors*

Aphelocoma wollweberi
Aquila chrysaetos
Aramus guarauna
Archilochus alexandri
Archilochus colubris
Ardea alba
Ardea herodias
Arenaria interpres
Arremonops rufivirgatus
Artemisiospiza belli
Artemisiospiza nevadensis
Asio flammeus
Asio otus
Athene cunicularia
Auriparus flaviceps
Aythya affinis
Aythya americana
Aythya collaris
Aythya marila
Aythya valisineria
Baeolophus atricristatus
Baeolophus bicolor
Baeolophus inornatus
Baeolophus ridgwayi
Baeolophus wollweberi
Bartramia longicauda
Bombycilla cedrorum
Bombycilla garrulus
Bonasa umbellus
Botaurus lentiginosus
Brachyramphus marmoratus
Branta bernicla
Branta canadensis
Branta hutchinsii
Bubo virginianus
Bubulcus ibis
Bucephala albeola
Bucephala clangula
Bucephala islandica
Buteo albonotatus
Buteo brachyurus
Buteo jamaicensis
Buteo lagopus
Buteo lineatus
Buteo plagiatus
Buteo platypterus

Anas fulvigula
Anas penelope
Anas platyrhynchos
Anas rubripes
Anas strepera
Anhinga anhinga
Anser albifrons
Anthracothorax prevostii
Anthus rubescens
Anthus spragueii
Antrostomus carolinensis
Antrostomus vociferus
Aphelocoma californica
Aphelocoma coerulescens
Aphelocoma wollweberi
Aquila chrysaetos
Ara ararauna
Ara severus
Aramus guarauna
Aratinga nenday
Aratinga weddellii
Archilochus alexandri
Archilochus colubris
Ardea alba
Ardea herodias
Ardenna bulleri
Ardenna carneipes
Ardenna creatopus
Ardenna gravis
Ardenna grisea
Ardenna tenuirostris
Arenaria interpres
Arenaria melanocephala
Arremonops rufivirgatus
Artemisiospiza belli
Artemisiospiza nevadensis
Asio flammeus
Asio otus
Athene cunicularia
Auriparus flaviceps
Aythya affinis
Aythya americana
Aythya collaris
Aythya marila
Aythya valisineria
Baeolophus atricristatus

Buteo regalis
Buteo swainsoni
Buteogallus anthracinus
Butorides striata
Cairina moschata
Calamospiza melanocorys
Calcarius lapponicus
Calcarius ornatus
Calcarius pictus
Calidris alba
Calidris bairdii
Calidris minutilla
Calidris pusilla
Calidris virgata
Callipepla californica
Callipepla gambelii
Callipepla squamata
Calothorax lucifer
Calypte anna
Calypte costae
Camptostoma imberbe
Campylorhynchus brunneicapillus
Caracara cheriway
Cardellina canadensis
Cardellina pusilla
Cardellina rubrifrons
Cardinalis cardinalis
Cardinalis sinuatus
Cathartes aura
Catharus bicknelli
Catharus fuscescens
Catharus guttatus
Catharus minimus
Catharus ustulatus
Catherpes mexicanus
Centrocercus minimus
Centrocercus urophasianus
Cepphus columba
Cepphus grylle
Cerorhinca monocerata
Certhia americana
Chaetura pelagica
Chaetura vauxi
Chamaea fasciata
Charadrius melodus
Charadrius montanus

Baeolophus bicolor
Baeolophus inornatus
Baeolophus ridgwayi
Baeolophus wollweberi
Bartramia longicauda
Basileuterus culicivorus
Basileuterus rufifrons
Bombycilla cedrorum
Bombycilla garrulus
Bonasa umbellus
Botaurus lentiginosus
Brachyramphus brevirostris
Brachyramphus marmoratus
Branta bernicla
Branta canadensis
Branta hutchinsii
Brotogeris chiriri
Brotogeris jugularis
Brotogeris versicolurus
Bubo scandiacus
Bubo virginianus
Bubulcus ibis
Bucephala albeola
Bucephala clangula
Bucephala islandica
Buteo albonotatus
Buteo brachyurus
Buteo jamaicensis
Buteo lagopus
Buteo lineatus
Buteo plagiatus
Buteo platypterus
Buteo regalis
Buteo swainsoni
Buteogallus anthracinus
Buteogallus urubitinga
Butorides virescens
Cairina moschata
Calamospiza melanocorys
Calcarius lapponicus
Calcarius ornatus
Calcarius pictus
Calidris acuminata
Calidris alba
Calidris alpina
Calidris bairdii

Charadrius nivosus
Charadrius semipalmatus
Charadrius wilsonia
Charadrius vociferus
Chlidonias niger
Chloroceryle americana
Chondestes grammacus
Chondrohierax uncinatus
Chordeiles acutipennis
Chordeiles minor
Cinclus mexicanus
Circus hudsonius
Cistothorus palustris
Cistothorus platensis
Clangula hyemalis
Coccyzus americanus
Coccyzus erythropthalmus
Coccyzus minor
Colaptes auratus
Colaptes chrysoides
Colinus virginianus
Columbina inca
Columbina passerina
Columbina talpacoti
Contopus cooperi
Contopus pertinax
Contopus sordidulus
Contopus virens
Coragyps atratus
Corvus brachyrhynchos
Corvus caurinus
Corvus corax
Corvus cryptoleucus
Corvus ossifragus
Coturnicops noveboracensis
Crotophaga ani
Crotophaga sulcirostris
Cyanocitta cristata
Cyanocitta stelleri
Cyanocorax yncas
Cygnus buccinator
Cygnus columbianus
Cynanthus latirostris
Cypseloides niger
Cyrtonyx montezumae
Dendragapus fuliginosus

Calidris canutus
Calidris fuscicollis
Calidris himantopus
Calidris maritima
Calidris mauri
Calidris melanotos
Calidris minutilla
Calidris ptilocnemis
Calidris pugnax
Calidris pusilla
Calidris subruficollis
Calidris virgata
Callipepla californica
Callipepla douglasii
Callipepla gambelii
Callipepla squamata
Calonectris diomedea
Calypte anna
Calypte costae
Camptostoma imberbe
Campylorhynchus brunneicapillus
Caracara cheriway
Cardellina canadensis
Cardellina pusilla
Cardinalis cardinalis
Cardinalis sinuatus
Cathartes aura
Catharus fuscescens
Catharus guttatus
Catharus minimus
Catharus ustulatus
Catherpes mexicanus
Centrocercus minimus
Centrocercus urophasianus
Cepphus columba
Cepphus grylle
Cerorhinca monocerata
Certhia americana
Chaetura pelagica
Chaetura vauxi
Chamaea fasciata
Charadrius melodus
Charadrius montanus
Charadrius nivosus
Charadrius semipalmatus
Charadrius wilsonia

Dendragapus obscurus
Dendrocygna autumnalis
Dendrocygna bicolor
Dolichonyx oryzivorus
Dryobates nuttallii
Dryobates pubescens
Dryobates scalaris
Dumetella carolinensis
Egretta caerulea
Egretta rufescens
Egretta thula
Egretta tricolor
Elanoides forficatus
Elanus leucurus
Empidonax alnorum
Empidonax difficilis
Empidonax flaviventris
Empidonax fulvifrons
Empidonax hammondii
Empidonax minimus
Empidonax oberholseri
Empidonax occidentalis
Empidonax traillii
Empidonax virescens
Empidonax wrightii
Eremophila alpestris
Eudocimus albus
Eugenes fulgens
Euphagus carolinus
Euphagus cyanocephalus
Falcipennis canadensis
Falco columbarius
Falco femoralis
Falco mexicanus
Falco peregrinus
Falco rusticolus
Falco sparverius
Fregata magnificens
Fulica americana
Gallinago delicata
Gallinula galeata
Gavia immer
Gavia pacifica
Gavia stellata
Gelochelidon nilotica
Geococcyx californianus
Charadrius vociferus
Chen caerulescens
Chen canagica
Chen rossii
Chlidonias niger
Chloroceryle americana
Chondestes grammacus
Chondrohierax uncinatus
Chordeiles acutipennis
Chordeiles minor
Chroicocephalus philadelphia
Chroicocephalus ridibundus
Cinclus mexicanus
Cistothorus palustris
Cistothorus platensis
Clangula hyemalis
Coccyzus americanus
Coccyzus erythropthalmus
Coccyzus minor
Coereba flaveola
Colaptes auratus
Colaptes chrysoides
Colinus virginianus
Columbina inca
Columbina passerina
Columbina talpacoti
Contopus cooperi
Contopus pertinax
Contopus sordidulus
Contopus virens
Coragyps atratus
Corvus brachyrhynchos
Corvus caurinus
Corvus corax
Corvus cryptoleucus
Corvus imparatus
Corvus ossifragus
Coturnicops noveboracensis
Crotophaga ani
Crotophaga sulcirostris
Cyanocitta cristata
Cyanocitta stelleri
Cyanocompsa parellina
Cyanocorax sanblasianus
Cyanocorax yncas
Cygnus buccinator

Geothlypis formosa
Geothlypis philadelphia
Geothlypis tolmiei
Geothlypis trichas
Geranoaetus albicaudatus
Glaucidium brasilianum
Glaucidium gnoma
Grus americana
Gymnogyps californianus
Gymnorhinus cyanocephalus
Haematopus ater
Haematopus palliatus
Haemorhous cassinii
Haemorhous mexicanus
Haemorhous purpureus
Haliaeetus leucocephalus
Helmitheros vermivorum
Hesperiphona vespertina
Himantopus himantopus
Hirundo rustica
Histrionicus histrionicus
Hydrobates leucorhous
Hydroprogne caspia
Hylatomus pileatus
Hylocichla mustelina
Icteria virens
Icterus bullockii
Icterus cucullatus
Icterus galbula
Icterus graduacauda
Icterus gularis
Icterus parisorum
Icterus pectoralis
Icterus spurius
Ictinia mississippiensis
Ixobrychus exilis
Ixoreus naevius
Junco hyemalis
Junco phaeonotus
Lagopus lagopus
Lagopus leucura
Lagopus muta
Lampornis clemenciae
Lanius borealis
Lanius ludovicianus
Larus atricilla
Cygnus columbianus
Cygnus cygnus
Cygnus melancoryphus
Cynanthus latirostris
Cypseloides niger
Cyrtonyx montezumae
Dendragapus fuliginosus
Dendragapus obscurus
Dendrocygna autumnalis
Dendrocygna bicolor
Dolichonyx oryzivorus
Dryocopus pileatus
Dumetella carolinensis
Egretta caerulea
Egretta rufescens
Egretta thula
Egretta tricolor
Elanoides forficatus
Elanus leucurus
Empidonax difficilis
Empidonax flaviventris
Empidonax hammondii
Empidonax minimus
Empidonax oberholseri
Empidonax occidentalis
Empidonax traillii
Empidonax virescens
Empidonax wrightii
Eremophila alpestris
Eudocimus albus
Eudocimus ruber
Eugenes fulgens
Euphagus carolinus
Euphagus cyanocephalus
Eupsittula canicularis
Euptilotis neoxenus
Falcipennis canadensis
Falco columbarius
Falco femoralis
Falco mexicanus
Falco peregrinus
Falco rusticolus
Falco sparverius
Fratercula arctica
Fratercula cirrhata
Fratercula corniculata

Larus californicus
Larus canus
Larus delawarensis
Larus glaucescens
Larus heermanni
Larus hyperboreus
Larus marinus
Larus occidentalis
Larus philadelphia
Larus pipixcan
Larus ridibundus
Larus smithsonianus
Laterallus jamaicensis
Leiothlypis celata
Leiothlypis luciae
Leiothlypis peregrina
Leiothlypis ruficapilla
Leiothlypis virginiae
Leptotila verreauxi
Leuconotopicus albolarvatus
Leuconotopicus arizonae
Leuconotopicus borealis
Leuconotopicus villosus
Leucosticte atrata
Leucosticte australis
Leucosticte tephrocotis
Limnodromus griseus
Limnodromus scolopaceus
Limnothlypis swainsonii
Limosa fedoa
Limosa haemastica
Lophodytes cucullatus
Loxia curvirostra
Loxia leucoptera
Mareca americana
Mareca strepera
Megaceryle alcyon
Megaceryle torquata
Megascops asio
Megascops kennicottii
Megascops trichopsis
Melanerpes aurifrons
Melanerpes carolinus
Melanerpes erythrocephalus
Melanerpes formicivorus
Melanerpes lewis
Fulica americana
Fulmarus glacialis
Gallinago delicata
Gallinula galeata
Gallus gallus
Gavia adamsii
Gavia immer
Gavia pacifica
Gavia stellata
Gelochelidon nilotica
Geococcyx californianus
Geothlypis formosa
Geothlypis philadelphia
Geothlypis poliocephala
Geothlypis tolmiei
Geothlypis trichas
Geranoaetus albicaudatus
Geranospiza caerulescens
Glaucidium brasilianum
Glaucidium gnoma
Grus americana
Grus canadensis
Gymnogyps californianus
Gymnorhinus cyanocephalus
Haematopus bachmani
Haematopus palliatus
Haemorhous cassinii
Haemorhous mexicanus
Haemorhous purpureus
Haliaeetus leucocephalus
Helmitheros vermivorum
Himantopus mexicanus
Hirundo rustica
Histrionicus histrionicus
Hydrocoloeus minutus
Hydroprogne caspia
Hylocharis leucotis
Hylocichla mustelina
Icteria virens
Icterus bullockii
Icterus cucullatus
Icterus galbula
Icterus graduacauda
Icterus gularis
Icterus parisorum
Icterus pectoralis

Melanerpes uropygialis
Melanitta americana
Melanitta fusca
Melanitta perspicillata
Meleagris gallopavo
Melospiza georgiana
Melospiza lincolnii
Melospiza melodia
Melozone aberti
Melozone crissalis
Melozone fusca
Mergus merganser
Mergus serrator
Micrathene whitneyi
Mimus gundlachii
Mimus polyglottos
Mniotilta varia
Molothrus aeneus
Molothrus ater
Molothrus bonariensis
Morus bassanus
Myadestes townsendi
Mycteria americana
Myiarchus cinerascens
Myiarchus crinitus
Myiarchus tuberculifer
Myiarchus tyrannulus
Myioborus pictus
Myiodynastes luteiventris
Nucifraga columbiana
Numenius americanus
Numenius phaeopus
Nyctanassa violacea
Nycticorax nycticorax
Nyctidromus albicollis
Oenanthe oenanthe
Onychoprion aleuticus
Oporornis agilis
Oreortyx pictus
Oreoscoptes montanus
Ortalis vetula
Oxyura jamaicensis
Pandion haliaetus
Parabuteo unicinctus
Parkesia motacilla
Parkesia noveboracensis
Icterus pustulatus
Icterus spurius
Icterus wagleri
Ictinia mississippiensis
Ixobrychus exilis
Ixoreus naevius
Jacana spinosa
Junco hyemalis
Junco phaeonotus
Lagopus lagopus
Lagopus leucura
Lagopus muta
Lampornis clemenciae
Lanius ludovicianus
Larus belcheri
Larus californicus
Larus canus
Larus delawarensis
Larus dominicanus
Larus fuscus
Larus glaucescens
Larus glaucoides
Larus heermanni
Larus hyperboreus
Larus livens
Larus marinus
Larus occidentalis
Larus schistisagus
Larus thayeri
Laterallus jamaicensis
Leptotila verreauxi
Leucophaeus atricilla
Leucophaeus modestus
Leucophaeus pipixcan
Leucosticte atrata
Leucosticte australis
Leucosticte tephrocotis
Limnodromus griseus
Limnodromus scolopaceus
Limnothlypis swainsonii
Limosa fedoa
Limosa haemastica
Limosa lapponica
Lophodytes cucullatus
Loxia leucoptera
Megaceryle alcyon

Passerculus bairdii
Passerculus henslowii
Passerculus sandwichensis
Passerella arborea
Passerella iliaca
Passerina amoena
Passerina caerulea
Passerina ciris
Passerina cyanea
Passerina versicolor
Patagioenas fasciata
Patagioenas flavirostris
Patagioenas leucocephala
Pelecanus erythrorhynchos
Pelecanus occidentalis
Perisoreus canadensis
Petrochelidon fulva
Petrochelidon pyrrhonota
Peucaea aestivalis
Peucaea botterii
Peucaea carpalis
Peucaea cassinii
Peucedramus taeniatus
Phainopepla nitens
Phalacrocorax auritus
Phalacrocorax brasilianus
Phalacrocorax carbo
Phalacrocorax pelagicus
Phalacrocorax penicillatus
Phalaenoptilus nuttallii
Phalaropus lobatus
Pheucticus ludovicianus
Pheucticus melanocephalus
Phylloscopus borealis
Pica hudsonia
Pica nutalli
Picoides arcticus
Picoides tridactylus
Pinicola enucleator
Pipilo chlorurus
Pipilo erythrophthalmus
Pipilo maculatus
Piranga ludoviciana
Piranga olivacea
Piranga rubra
Pitangus sulphuratus
Megaceryle torquata
Megascops asio
Megascops kennicottii
Megascops trichopsis
Melanerpes aurifrons
Melanerpes carolinus
Melanerpes erythrocephalus
Melanerpes formicivorus
Melanerpes lewis
Melanerpes uropygialis
Melanotis caerulescens
Meleagris gallopavo
Melozone aberti
Melozone crissalis
Melozone fusca
Mergus serrator
Micrathene whitneyi
Milvago chimachima
Mimus gundlachii
Mitrephanes phaeocercus
Molothrus aeneus
Molothrus bonariensis
Morus bassanus
Myadestes townsendi
Mycteria americana
Myiarchus cinerascens
Myiarchus crinitus
Myiarchus nuttingi
Myiarchus sagrae
Myiarchus tuberculifer
Myiarchus tyrannulus
Myioborus pictus
Myiodynastes luteiventris
Myiopsitta monachus
Nomonyx dominicus
Nucifraga columbiana
Numenius americanus
Numenius phaeopus
Nyctanassa violacea
Nycticorax nycticorax
Nyctidromus albicollis
Oceanites oceanicus
Oceanodroma furcata
Oceanodroma homochroa
Oceanodroma leucorhoa
Oceanodroma melania

Platalea ajaja
Plegadis chihi
Plegadis falcinellus
Pluvialis dominica
Podiceps grisegena
Podiceps nigricollis
Podilymbus podiceps
Poecile atricapillus
Poecile carolinensis
Poecile gambeli
Poecile hudsonicus
Poecile rufescens
Poecile sclateri
Polioptila caerulea
Polioptila californica
Polioptila melanura
Pooecetes gramineus
Porphyrio martinicus
Porzana carolina
Progne subis
Protonotaria citrea
Psaltriparus minimus
Psiloscops flammeolus
Ptychoramphus aleuticus
Pyrocephalus rubinus
Quiscalus major
Quiscalus mexicanus
Quiscalus quiscula
Rallus crepitans
Rallus elegans
Rallus limicola
Rallus obsoletus
Recurvirostra americana
Regulus calendula
Regulus satrapa
Rhynchophanes mccownii
Riparia riparia
Rissa tridactyla
Rostrhamus sociabilis
Rynchops niger
Salpinctes obsoletus
Sayornis nigricans
Sayornis phoebe
Sayornis saya
Scolopax minor
Seiurus aurocapilla
Onychoprion anaethetus
Onychoprion fuscatus
Oporornis agilis
Oreortyx pictus
Oreoscoptes montanus
Oreothlypis celata
Oreothlypis luciae
Oreothlypis peregrina
Oreothlypis ruficapilla
Oreothlypis superciliosa
Oreothlypis virginiae
Ortalis vetula
Oxyura jamaicensis
Pachyramphus aglaiae
Pagophila eburnea
Pandion haliaetus
Parabuteo unicinctus
Parkesia motacilla
Parkesia noveboracensis
Paroaria coronata
Passerculus sandwichensis
Passerella iliaca
Passerina amoena
Passerina caerulea
Passerina ciris
Passerina cyanea
Passerina versicolor
Patagioenas fasciata
Patagioenas flavirostris
Patagioenas leucocephala
Pelecanus erythrorhynchos
Pelecanus occidentalis
Perisoreus canadensis
Petrochelidon fulva
Petrochelidon pyrrhonota
Peucaea aestivalis
Peucaea botterii
Peucaea carpalis
Peucaea cassinii
Peucedramus taeniatus
Phainopepla nitens
Phalacrocorax auritus
Phalacrocorax brasilianus
Phalacrocorax carbo
Phalacrocorax pelagicus
Phalacrocorax penicillatus

Selasphorus calliope
Selasphorus platycercus
Selasphorus rufus
Selasphorus sasin
Setophaga americana
Setophaga caerulescens
Setophaga castanea
Setophaga cerulea
Setophaga chrysoparia
Setophaga citrina
Setophaga coronata
Setophaga discolor
Setophaga dominica
Setophaga fusca
Setophaga graciae
Setophaga kirtlandii
Setophaga magnolia
Setophaga nigrescens
Setophaga occidentalis
Setophaga palmarum
Setophaga pensylvanica
Setophaga petechia
Setophaga pinus
Setophaga pitiayumi
Setophaga ruticilla
Setophaga striata
Setophaga tigrina
Setophaga townsendi
Setophaga virens
Sialia currucoides
Sialia mexicana
Sialia sialis
Sitta canadensis
Sitta carolinensis
Sitta pusilla
Sitta pygmaea
Somateria mollissima
Spatula clypeata
Spatula cyanoptera
Spatula discors
Sphyrapicus nuchalis
Sphyrapicus ruber
Sphyrapicus thyroideus
Sphyrapicus varius
Spinus lawrencei
Spinus pinus
Phalacrocorax urile
Phalaenoptilus nuttallii
Phalaropus fulicarius
Phalaropus lobatus
Phalaropus tricolor
Pheucticus ludovicianus
Pheucticus melanocephalus
Phoebastria albatrus
Phoebastria immutabilis
Phoebastria nigripes
Phoenicopterus ruber
Pica hudsonia
Picoides albolarvatus
Picoides arcticus
Picoides arizonae
Picoides borealis
Picoides dorsalis
Picoides nuttallii
Picoides pubescens
Picoides scalaris
Picoides stricklandi
Picoides villosus
Pinicola enucleator
Pionus maximiliani
Pionus senilis
Pipilo chlorurus
Pipilo erythrophthalmus
Pipilo maculatus
Piranga flava
Piranga ludoviciana
Piranga olivacea
Piranga rubra
Pitangus sulphuratus
Platalea ajaja
Plectrophenax hyperboreus
Plectrophenax nivalis
Plegadis chihi
Plegadis falcinellus
Pluvialis dominica
Pluvialis fulva
Pluvialis squatarola
Podiceps grisegena
Podiceps nigricollis
Podilymbus podiceps
Poecile atricapillus
Poecile carolinensis

Spinus psaltria
Spinus tristis
Spiza americana
Spizella atrogularis
Spizella breweri
Spizella pallida
Spizella passerina
Spizella pusilla
Steganopus tricolor
Stelgidopteryx serripennis
Stercorarius longicaudus
Stercorarius parasiticus
Sterna forsteri
Sterna hirundo
Sterna paradisaea
Sternula antillarum
Strix nebulosa
Strix occidentalis
Strix varia
Sturnella magna
Sturnella neglecta
Sula dactylatra
Sula leucogaster
Sula nebouxii
Surnia ulula
Tachybaptus dominicus
Tachycineta bicolor
Tachycineta thalassina
Thalasseus elegans
Thalasseus maximus
Thalasseus sandvicensis
Thryomanes bewickii
Thryophilus sinaloa
Thryothorus ludovicianus
Toxostoma bendirei
Toxostoma crissale
Toxostoma curvirostre
Toxostoma lecontei
Toxostoma longirostre
Toxostoma redivivum
Toxostoma rufum
Tringa flavipes
Tringa incana
Tringa melanoleuca
Tringa semipalmata
Tringa solitaria
Poecile cinctus
Poecile gambeli
Poecile hudsonicus
Poecile rufescens
Poecile sclateri
Polioptila caerulea
Polioptila californica
Polioptila melanura
Polioptila nigriceps
Polysticta stelleri
Pooecetes gramineus
Porphyrio martinicus
Porzana carolina
Progne subis
Protonotaria citrea
Psaltriparus minimus
Psiloscops flammeolus
Psittacara chloropterus
Psittacara erythrogenys
Psittacara finschi
Psittacara holochlorus
Psittacara leucophthalmus
Psittacara mitratus
Psittacara wagleri
Ptychoramphus aleuticus
Pyrocephalus rubinus
Pyrrhura frontalis
Pyrrhura molinae
Quiscalus major
Quiscalus mexicanus
Quiscalus quiscula
Rallus crepitans
Rallus elegans
Rallus limicola
Rallus obsoletus
Recurvirostra americana
Regulus satrapa
Rhodostethia rosea
Rhynchophanes mccownii
Rhynchopsitta pachyrhyncha
Riparia riparia
Rissa tridactyla
Rostrhamus sociabilis
Rupornis magnirostris
Rynchops niger
Salpinctes obsoletus

Troglodytes aedon
Troglodytes hiemalis
Troglodytes pacificus
Trogon elegans
Turdus migratorius
Tympanuchus cupido
Tympanuchus pallidicinctus
Tympanuchus phasianellus
Tyrannus couchii
Tyrannus crassirostris
Tyrannus dominicensis
Tyrannus forficatus
Tyrannus melancholicus
Tyrannus tyrannus
Tyrannus verticalis
Tyrannus vociferans
Tyto alba
Uria aalge
Vermivora chrysoptera
Vermivora cyanoptera
Vireo altiloquus
Vireo atricapilla
Vireo bellii
Vireo cassinii
Vireo flavifrons
Vireo gilvus
Vireo griseus
Vireo huttoni
Vireo olivaceus
Vireo philadelphicus
Vireo plumbeus
Vireo solitarius
Vireo vicinior
Xanthocephalus xanthocephalus
Zenaida asiatica
Zenaida macroura
Zonotrichia albicollis
Zonotrichia atricapilla
Zonotrichia leucophrys
Zonotrichia querula
Sayornis nigricans
Sayornis phoebe
Sayornis saya
Scolopax minor
Seiurus aurocapilla
Selasphorus calliope
Selasphorus platycercus
Selasphorus rufus
Selasphorus sasin
Setophaga americana
Setophaga caerulescens
Setophaga castanea
Setophaga cerulea
Setophaga chrysoparia
Setophaga citrina
Setophaga coronata
Setophaga discolor
Setophaga dominica
Setophaga fusca
Setophaga graciae
Setophaga magnolia
Setophaga nigrescens
Setophaga occidentalis
Setophaga palmarum
Setophaga pensylvanica
Setophaga petechia
Setophaga pinus
Setophaga pitiayumi
Setophaga ruticilla
Setophaga striata
Setophaga tigrina
Setophaga townsendi
Setophaga virens
Sialia currucoides
Sialia mexicana
Sialia sialis
Sitta canadensis
Sitta carolinensis
Sitta pusilla
Sitta pygmaea
Somateria mollissima
Somateria spectabilis
Sphyrapicus nuchalis
Sphyrapicus ruber
Sphyrapicus thyroideus
Sphyrapicus varius

Spindalis zena
Spinus lawrencei
Spinus pinus
Spinus psaltria
Spinus tristis
Spiza americana
Spizella atrogularis
Spizella breweri
Spizella pallida
Spizella passerina
Spizella pusilla
Spizelloides arborea
Sporophila torqueola
Stelgidopteryx serripennis
Stercorarius maccormicki
Stercorarius parasiticus
Stercorarius pomarinus
Stercorarius skua
Sternula antillarum
Streptoprocne zonaris
Strix nebulosa
Strix occidentalis
Strix varia
Sturnella magna
Sturnella neglecta
Sula dactylatra
Sula leucogaster
Sula nebouxii
Sula sula
Surnia ulula
Synthliboramphus antiquus
Synthliboramphus craveri
Synthliboramphus scrippsi
Tachybaptus dominicus
Tachycineta bicolor
Tachycineta thalassina
Thalasseus elegans
Thalasseus maximus
Thalasseus sandvicensis
Thectocercus acuticaudatus
Thryomanes bewickii
Thryophilus sinaloa
Thryothorus ludovicianus
Tigrisoma mexicanum
Toxostoma bendirei
Toxostoma crissale

Toxostoma curvirostre
Toxostoma lecontei
Toxostoma longirostre
Toxostoma redivivum
Toxostoma rufum
Tringa flavipes
Tringa incana
Tringa melanoleuca
Tringa semipalmata
Tringa solitaria
Troglodytes aedon
Troglodytes hiemalis
Troglodytes pacificus
Trogon elegans
Turdus assimilis
Turdus grayi
Turdus migratorius
Turdus rufopalliatus
Tympanuchus cupido
Tympanuchus pallidicinctus
Tympanuchus phasianellus
Tyrannus caudifasciatus
Tyrannus couchii
Tyrannus crassirostris
Tyrannus dominicensis
Tyrannus forficatus
Tyrannus melancholicus
Tyrannus savana
Tyrannus tyrannus
Tyrannus verticalis
Tyrannus vociferans
Tyto alba
Uria aalge
Uria lomvia
Vermivora chrysoptera
Vermivora cyanoptera
Vireo altiloquus
Vireo atricapilla
Vireo bellii
Vireo cassinii
Vireo crassirostris
Vireo flavifrons
Vireo flavoviridis
Vireo gilvus
Vireo griseus
Vireo huttoni

Vireo olivaceus
Vireo philadelphicus
Vireo plumbeus
Vireo solitarius
Vireo vicinior
Xanthocephalus xanthocephalus
Xema sabini
Zenaida asiatica
Zenaida aurita
Zenaida macroura
Zonotrichia albicollis
Zonotrichia atricapilla
Zonotrichia leucophrys
Zonotrichia querula
